# Complimentary action of structured and unstructured domains of epsin supports clathrin-mediated endocytosis at high tension

**DOI:** 10.1038/s42003-020-01471-6

**Published:** 2020-12-08

**Authors:** Jophin G. Joseph, Carlos Osorio, Vivian Yee, Ashutosh Agrawal, Allen P. Liu

**Affiliations:** 1 Department of Mechanical Engineering, University of Michigan, Ann Arbor, MI USA; 2 Department of Mechanical Engineering, University of Houston, Houston, TX USA; 3 Department of Biomedical Engineering, University of Michigan, Ann Arbor, MI USA; 4 Cellular and Molecular Biology Program, University of Michigan, Ann Arbor, MI USA; 5 Department of Biophysics, University of Michigan, Ann Arbor, MI USA

**Keywords:** Membrane trafficking, Endocytosis

## Abstract

Membrane tension plays an inhibitory role in clathrin-mediated endocytosis (CME) by impeding the transition of flat plasma membrane to hemispherical clathrin-coated structures (CCSs). Membrane tension also impedes the transition of hemispherical domes to omega-shaped CCSs. However, CME is not completely halted in cells under high tension conditions. Here we find that epsin, a membrane bending protein which inserts its N-terminus H_0_ helix into lipid bilayer, supports flat-to-dome transition of a CCS and stabilizes its curvature at high tension. This discovery is supported by molecular dynamic simulation of the epsin N-terminal homology (ENTH) domain that becomes more structured when embedded in a lipid bilayer. In addition, epsin has an intrinsically disordered protein (IDP) C-terminus domain which induces membrane curvature via steric repulsion. Insertion of H_0_ helix into lipid bilayer is not sufficient for stable epsin recruitment. Epsin’s binding to adaptor protein 2 and clathrin is critical for epsin’s association with CCSs under high tension conditions, supporting the importance of multivalent interactions in CCSs. Together, our results support a model where the ENTH and unstructured IDP region of epsin have complementary roles to ensure CME initiation and CCS maturation are unimpeded under high tension environments.

## Abstract (web summary)

Using imaging and molecular dynamics simulations, Joseph et al characterize the tension-responsive recruitment of the membrane bending protein epsin and its stabilization of clathrin-coated structures (CCSs) to form clathrin-coated pits. They find epsin’s ENTH- and unstructured IDP domains play complementary roles in CCS maturation under high tension.

## Introduction

Clathrin-mediated endocytosis (CME) involves the internalization of cargo by sculpting plasma membrane into 60–120 nm-sized buds, supported by a clathrin protein coat^1^. CME is a well-studied endocytic pathway present in organisms at all developmental stages^1–4^. It plays a critical role in nutrient uptake, intracellular trafficking, and signal transduction^1,3^. CME is a multistep process involving (i) initiation of membrane budding with adapter proteins and membrane bending proteins^5–7^, (ii) clathrin-coat formation^8,9^, (iii) maturation of coated pits^10–12^, and (iv) dynamin-mediated scission of the buds^1,3,7^. Progression of CME involves extensive deformation of the flat plasma membrane to Ω-shaped pits^1,12^. Given CME is a mechanical process, membrane tension has been shown to play an inhibitory role during the membrane deformation process preventing the transition from a flat membrane to hemispherical domes^10,11^ and the transition from hemispherical domes to Ω-shaped pits^10–12^. Yet, CME is observed ubiquitously in cells under different membrane tension regimes and this points to the existence of tension-sensitive molecular mechanisms supporting CME^12–14^. The actin-mediated transition of hemispherical domes to Ω-shaped pits at high tension was established by Boulant et al.^12,15^. However, how membrane-associated proteins aid to overcome the elevated energy barrier needed to initiate budding remains an open question. We hypothesize that endocytic membrane bending proteins possess the ability to sense and counteract membrane tension to facilitate clathrin-coat budding at elevated tension.

Epsin/AP180 family is a major family of proteins involved in membrane bending during the initiation of CME^5,16,17^. Epsin, a prominent member of the family, is known to insert its N-terminus amphipathic helix (H_0_ helix in epsin N-terminus homology (ENTH) domain) into the bilayer with a wedging effect after binding to PIP_2_ to initiate membrane bending^16^. Epsin also has a C-terminus intrinsically disordered protein (IDP) region containing multiple binding sites to endocytic constituent proteins like clathrin and AP2 which stably dock epsin in the endocytic pit^18–21^. An alternate hypothesis proposed recently posits the C-terminus IDP domain of epsin initiates membrane bending via steric crowding^22,23^. In vitro studies have shown that the insertion of purified ENTH into giant unilamellar vesicles (GUVs) reduces membrane rigidity and area compressibility modulus of the lipid bilayer^24^. Further, the recruitment of ENTH softens the bilayer at high tension and initiates tubulation at low tension^25^. ENTH is shown to recruit selectively to the highly curved surface of cylindrical membrane tethers held at different tension^26^. An increase in lipid packing defects at high tension may be key in aiding helix insertion at high tension from theoretical studies^27,28^. This evidence points to the existence of a tension-sensitive recruitment mechanism of ENTH domain-containing proteins. There exist an interplay between membrane tension and peripheral protein density that mediates membrane deformation^29^. However, there is still ambiguity in the exact mechanism of ENTH binding at different tensions, and a lack of experimental evidence for tension-mediated recruitment of epsin to clathrin-coat nucleation sites in cells. Here, we used the combination of total internal reflection fluorescence (TIRF) and structured illumination microscopy (SIM) imaging, mechano-manipulation techniques, automated image analysis, and molecular dynamics (MD) simulation to investigate the tension-responsive recruitment of epsin and its stabilization of clathrin-coated structures (CCSs) to form clathrin-coated pits (CCPs). We demonstrated that the recruitment of epsin increases with membrane tension in CCSs. Further, using MD simulations and experiments involving epsin mutants, we deciphered the role of the H_0_ helix in tension sensing and the molecular mechanism of tension-mediated recruitment of epsin. In addition, we also demonstrate the role of the IDP domain of epsin in stabilizing CCSs in high-tension environments. Our work establishes a mechanism involving complementary roles of ENTH and IDP domains of epsin in recruitment into and stabilization of CCSs in a high-tension environment.

## Results

### Overexpression of epsin in cells reduces abortive CCSs at high tension

**Fig. 1:**
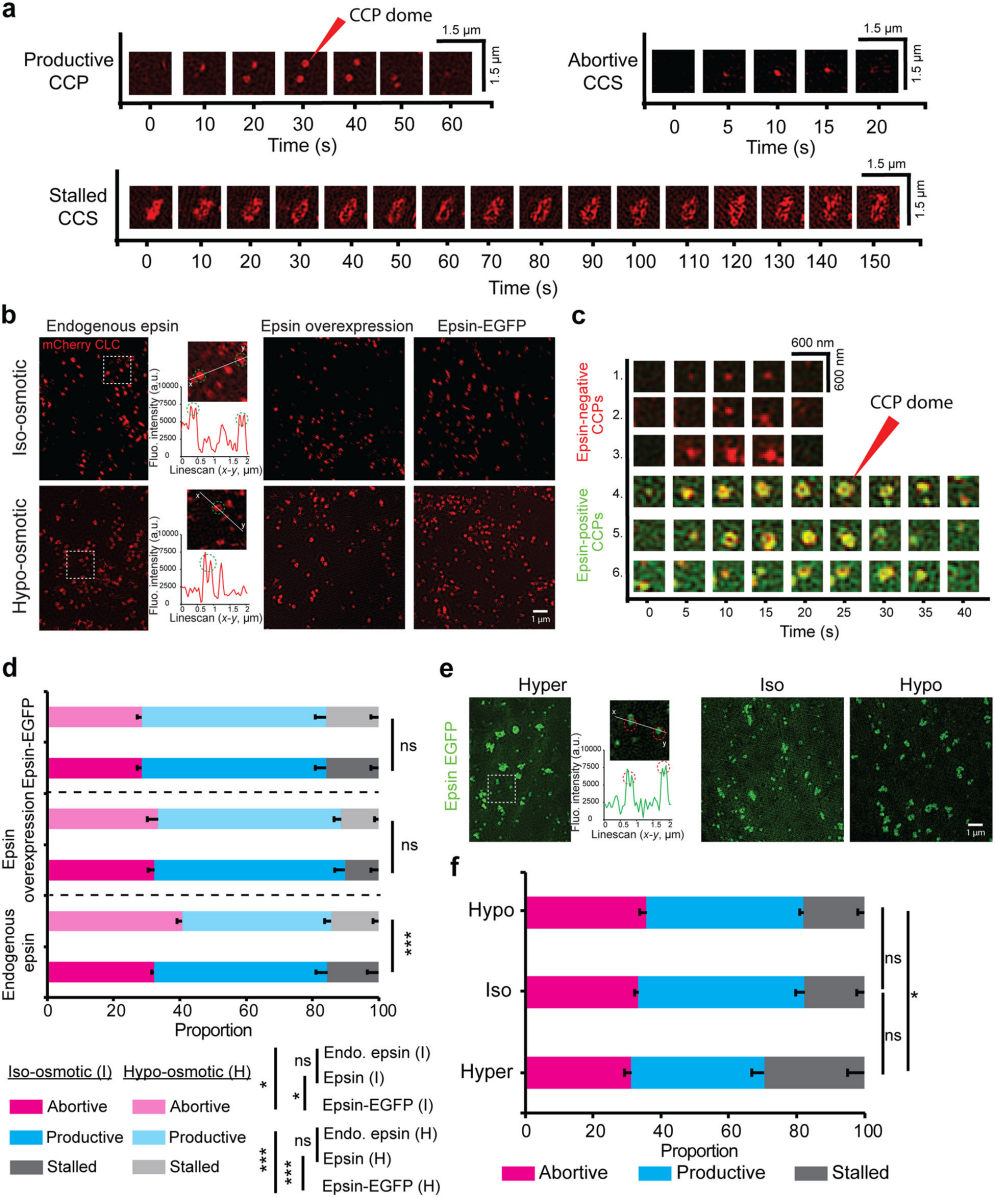
Overexpression of epsin in RPE cells reduces the population of abortive CCSs at high tension. **a** Lifetime montage of CCS/CCP (marker: mCherry CLC) classified as abortive, productive, and stalled imaged using SIM-TIRF. The formation of a CCS dome (manifested as a ring in SIM-TIRF) is shown with a red arrow. **b** SIM-TIRF images of clathrin in RPE cells expressing epsin at endogenous levels, overexpressing epsin, and overexpressing epsin EGFP under iso-osmotic (control—290 mmol/kg) and hypo-osmotic (220 mmol/kg) conditions. The formation of a CCS dome (highlighted by green circles) is depicted using intensity line scans. **c** Lifetime montage of CCSs (marker: mCherry CLC) with or without recruitment of epsin (green) using dual-color SIM-TIRF. **d** Percentage of abortive, productive, and stalled CCPs/CCSs in cells expressing endogenous epsin, overexpressing epsin, and overexpressing epsin EGFP under iso-osmotic (control) and hypo-osmotic conditions. **e** SIM-TIRF images of epsin EGFP in RPE cells under hyper- (440 mmol/kg), iso- (290 mmol/kg), and hypo-osmotic (220 mmol/kg) conditions. The formation of a CCS dome (highlighted by red circles) is depicted using an intensity line scan. **f** Percentage of abortive, productive, and stalled epsin EGFP structures under hyper-, iso-, and hypo-osmotic conditions. For **d**, the number of CCSs analyzed for endogenous epsin, epsin overexpression, and epsin EGFP overexpression were 26,240 (iso) and 16,918 (hypo), 17,914 (iso) and 14,722 (hypo), 31,776 (iso) and 41,949 (hypo), respectively taken from 6 cells under each condition. For **f**, the number of epsin EGFP structures analyzed were 31,404 (hyper), 36,536 (iso), and 31,085 (hypo), 31,776 (iso), respectively, taken from 6 cells under each condition. The error bars denote standard deviation. NS denotes not significant. *, **, *** represent *p* < 0.05, *p* < 0.01, and *p* < 0.001, respectively.

Previous work from our lab has shown that retinal pigment epithelial (RPE) cells spread on large fibronectin islands (to induce high membrane tension) exhibited an increase in the proportion of abortive CCSs and smaller CCPs^30,31^. Using SIM-TIRF super-resolution imaging of RPE cells stably expressing mCherry-clathrin light chain (CLC), we characterized the morphology of CCSs and classified them into three categories: (i) abortive CCSs, which are coated structures which dissemble before they reach maturation^32–34^, (ii) productive CCPs, which are coated structures that undergo initiation, assembly, and transition to coated-pits followed by membrane scission and internalization, and (iii) stalled CCSs, which are persistent, non-internalizing coated structures in the imaging field^8,12^. Lifetime analyses of CCSs have shown that abortive CCSs have a lifetime less than 20 s^35–37^, and stalled CCSs have a lifetime above 120 s^8,12,35^. Under the SIM-TIRF field, a matured CCS reaches a domed shape, manifested as a ring (Fig. 1a, shown with red arrow) to form a productive CCP^38^. The track of a productive CCP shows the evolution of a point signal into a ring structure, subsequently transitioned to a point signal, and finally disappeared from the SIM-TIRF field (Fig. 1a). By comparison, an abortive CCS did not transition from a point signal to a ring structure and disappeared from the SIM-TIRF field within a short time. Stalled CCSs were often clusters of CCSs in the shape of interconnected rings, which remained persistently in the SIM-TIRF field.

RPE cells expressing epsin at endogenous level, overexpressing epsin, and overexpressing epsin EGFP were imaged under iso-osmotic (control—290 mmol/kg osmolarity) and hypo-osmotic conditions (high tension—220 mmol/kg osmolarity) using SIM-TIRF (Fig. 1b). Hyper- and hypo-osmotic shock led to a reduction or increase in cell volume, as evident from the 3D cross-section of the cells reconstructed from confocal z stacks (Supplementary Figure 1a). Micropipette aspiration assay revealed that membrane aspiration into micropipette reduced from hyper- to iso- to hypo-osmotic condition (Supplementary Fig. 1b–d), similar to what was previously reported^39^. RPE cells have a low endogenous expression of epsin compared to cell lines like HEK, hence overexpression of epsin WT or epsin EGFP in RPE cells was used (Supplementary Fig. 2a). Dual-color SIM-TIRF imaging was used to identify the co-localization of epsin and clathrin in CCSs (Fig. 1c). The cells were imaged between 5 and 35 min after adding the new osmotic media. This is to ensure that the imaging was performed before the cells re-equilibrate their shape^8,40^. Cells under iso-osmotic conditions showed similar proportions of abortive CCSs irrespective of epsin expression level (Fig. 1d). However, overexpressing epsin-EGFP led to a reduction in the percentage of abortive CCSs compared to endogenous expression or overexpression of WT epsin under iso-osmotic conditions. On the other hand, when membrane tension was increased under hypo-osmotic conditions, cells expressing the endogenous levels of epsin showed an appreciable increase in abortive CCSs, consistent with previous findings^30^. In contrast, overexpression of WT epsin or epsin EGFP both maintained the same percentages of abortive CCSs under hypo-osmotic conditions compared to iso-osmotic conditions (Fig. 1d). Interestingly, cells overexpressing epsin EGFP had a higher percentage of stalled CCSs compared to cells overexpressing WT epsin. As a result, overexpression of WT epsin had a larger fraction of productive CCPs compared to epsin EGFP overexpression. To confirm the effect of epsin expression on the productiveness of CCPs, we performed transferrin-uptake assay in iso- and hypo-osmotic conditions for RPE cells expressing endogenous level, overexpressing epsin, and overexpressing epsin EGFP. Normalized transferrin uptake reduced with increased membrane tension increase in RPE cells expressing epsin at endogenous level, whereas both overexpression of epsin WT and epsin EGFP showed no significant changes with an increase in membrane tension (Supplementary Fig. 2b). The uptake of transferrin in iso- and hypo-osmotic conditions for RPE cells with endogenous level epsin, overexpressing epsin and overexpressing epsin EGFP closely correlated with the density of productive CCPs (Supplementary Fig. 2c). Further, membrane-associated structures with epsin EGFP were imaged at hyper- (low tension—440 mmol/kg osmolarity), iso- (control—290 mmol/kg osmolarity) and hypo- (high tension—220 mmol/kg osmolarity) osmotic conditions (Fig. 1e). As membrane tension increased, the percentage of abortive structures with epsin recruitment increased only slightly (Fig. 1f). However, at low tension, epsin recruitment resulted in CCS clustering and stalling (Fig. 1f). This stalling and clustering is consistent with in vitro observation of epsin tubulation at low tension in GUVs^22–25^. Even though clusters of CCSs which are stalled remained in the TIRF field during the acquisition period, they showed dynamic rearrangement of CCS rings including merging and splitting of rings (Supplementary Movie 1). Some clusters showed the disappearance of CCSs from the boundary of the stalled clusters (Supplementary Movie 1).

### Epsin recruitment increases as resting membrane tension increases

Epsin is an endocytic adapter protein recruited to the plasma membrane during initiation of CME to aid in membrane curvature generation^16^. The ability for pits to maintain stable curvatures until they reach maturation is essential for the successful completion of a CCS lifecycle^12^. Membrane tension is shown to counteract the stabilization of coated pits by increasing the energy required to form a pit^11^. To investigate whether epsin recruitment can counteract varying membrane tension, we manipulated resting or acute membrane tension of RPE cells co-expressing epsin EGFP and mCherry CLC by controlling cell spreading or by applying osmotic shock, respectively.

**Fig. 2:**
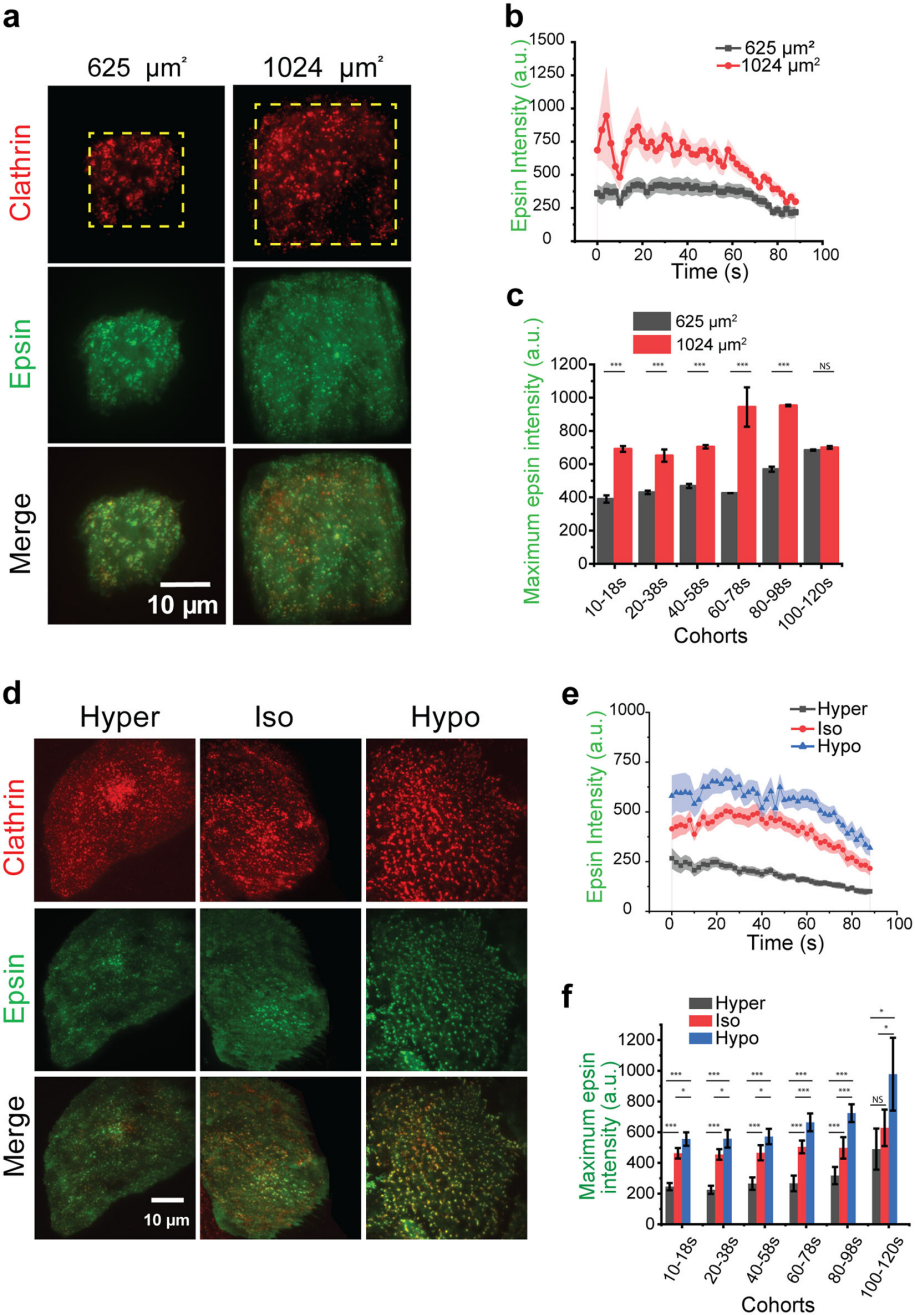
Increased tension increases recruitment of epsin into CCSs. **a** RPE cells expressing epsin EGFP and mCherry CLC spread on fibronectin square islands of size 25 and 32 µm. The resting membrane tension is higher for 32 µm islands. **b** The average intensity trace of epsin on 25 µm (black) and 32 µm (red) islands for CCSs with 60–78 s lifetime. **c** The average plateau intensity of epsin across different CCS lifetime cohorts for cells on 25 µm (black) and 32 µm (red) islands. **d** Fluorescence images of clathrin and epsin of RPE cells expressing epsin EGFP under different osmotic conditions. **e** The average plateau intensity of epsin in different osmotic media for CCSs with 60–78 s lifetime. **f** The average plateau intensity of epsin across different CCS lifetime cohorts. For **b**, **c**, N_cells_ for 25 µm square and N_cells_ for 32 µm square are 18 (N_tracks_ = 17,534) and 19 (N_tracks_ = 30,188), respectively. For **e**, **f**, the number of cells for hyper-, iso-, and hypo-osmotic conditions were 19 (N_tracks_ = 38282), 19 (N_tracks_ = 58,574), and 16 (N_tracks_ = 32,644), respectively. The error bars denote standard error. NS denotes not significant. *, **, *** represent *p* < 0.05, *p* < 0.01, and *p* < 0.001, respectively.

The resting tension of an adherent cell is related to the membrane tension exerted when the cell is fully spread^41,42^. The resting membrane tension of the cells was controlled by restricting cell spreading on microcontact-printed fibronectin islands of area 625 or 1024 µm^2^ (Fig. 2a). Cells spread on a larger area experienced higher membrane tension^30,41^. Using dual-color TIRF imaging and an automated algorithm^35^, we detected and tracked CCSs and quantified the lifetime, composition (whether both epsin and CLC are present or not), and intensity of fluorescently tagged epsin (EGFP) and CLC (mCherry) in CCSs. Cells imaged had similar expression levels of fluorescent proteins (epsin EGFP and mCherry CLC) between fibronectin islands of area 625^2^ or 1024 µm^2^ (Supplementary Fig. 3a). The intensity traces of CCSs belonging to the 60–78 s lifetime cohort showed that the intensity of EGFP epsin is higher in highly spread cells, suggesting epsin recruitment increases with an increase in resting tension (Fig. 2b). Interestingly, epsin intensity is already elevated at time 0 when clathrin detection began, suggesting epsin EGFP recruitment may precede clathrin recruitment. Further, the average plateau intensity (i.e., the maximum intensity during a coated pit’s lifetime) of epsin in other lifetime cohorts (from 10–18 to 80–98 s) also showed higher recruitment of epsin to CCSs in more spread cells in comparison with the less spread cells (Fig. 2c). In contrast, the intensity of mCherry CLC in the 80–98 s lifetime cohort and other lifetime cohorts had a small reduction as resting tension increased (Supplementary Fig. 3b, c), in agreement with a previous finding^30^. The ratio of epsin to clathrin also increased as the area of fibronectin islands increased (Supplementary Fig. 3d). Together, these results indicate that epsin recruitment per clathrin in CCSs increases with increasing resting tension.

### Epsin recruitment increases as acute membrane tension increases

**Fig. 3:**
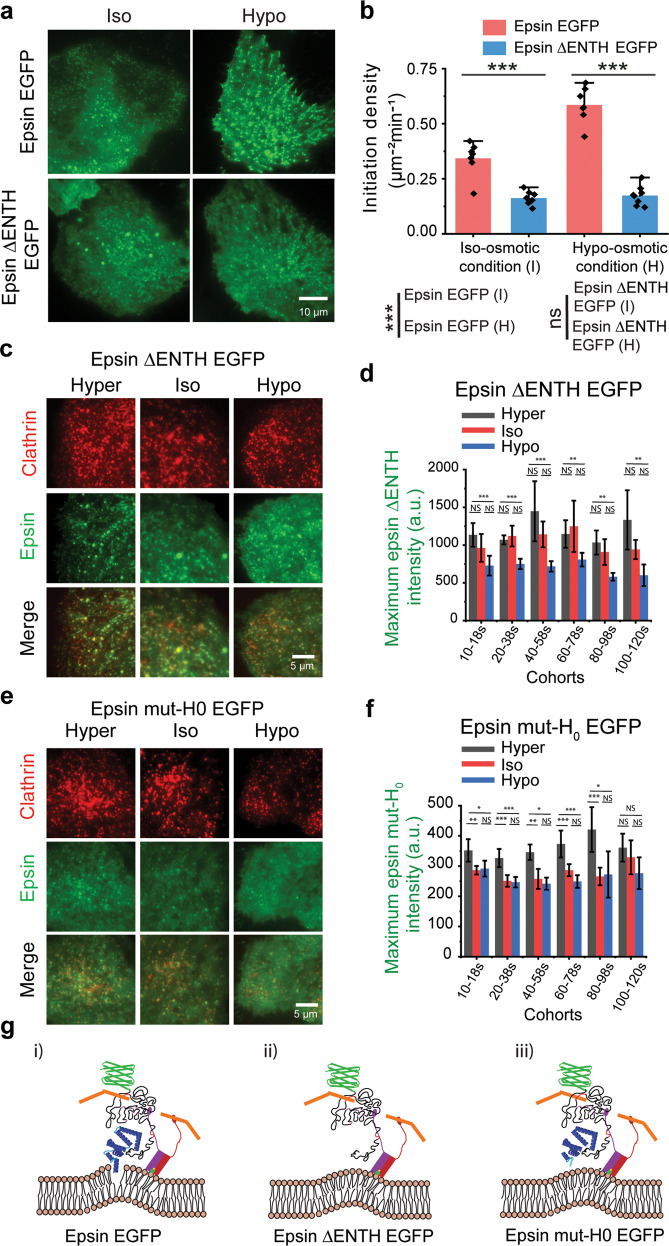
ENTH domain-containing H_0_ helix supports tension-mediated nucleation of epsin at CCS sites. **a** Fluorescence images of epsin, epsin ΔENTH at control (iso-) and high tension (hypo-osmotic conditions). **b** Initiation density of epsin puncta in epsin EGFP (salmon) expressing and epsin ΔENTH (blue) expressing cells under control (iso-osmotic) and high-tension (hypo-osmotic) conditions. **c** Representative fluorescence images of clathrin and epsin of RPE cells expressing epsin ΔENTH EGFP under different osmotic conditions. **d** The average plateau intensity of epsin ΔENTH in different CCS lifetime cohorts in different osmotic conditions. **e** Representative fluorescence images of clathrin and epsin of RPE cells expressing epsin mut-H_0_ EGFP under different osmotic shock conditions. **f** The average plateau intensity of epsin mut-H_0_ in different CCS lifetime cohorts in different osmotic conditions. **g** Proposed model for (i) epsin EGFP, (ii) epsin ΔENTH EGFP, and (iii) epsin mut-H_0_ EGFP nucleation at CCS sites. For **b**, N_cells_ expressing epsin EGFP under iso- and hypo-osmotic conditions were 7 (N_tracks_ = 21,570) and 7 (N_tracks_ = 24,611) respectively, and N_cells_ expressing epsin ΔENTH under iso- and hypo-osmotic conditions were 8 (N_tracks_ = 13,400) and 8 (N_tracks_ = 21,300), respectively. The N_cells_ expressing epsin ΔENTH EGFP for hyper-, iso-, and hypo-osmotic conditions in (**d**) were 12 (N_tracks_ = 19,328), 12 (N_tracks_ = 20,078), and 12 (N_tracks_ = 31,952), respectively. The N_cells_ expressing epsin mut-H_0_ for hyper-, iso-, and hypo-osmotic conditions in (**f**) were 18 (N_tracks_ = 32,327), 19 (N_tracks_ = 33,354), and 18 (N_tracks_ = 40,602), respectively. The error bars denote standard error. NS denotes not significant. *, **, *** represent *p* < 0.05, *p* < 0.01, and *p* < 0.001, respectively.

To investigate whether epsin shows a similar recruitment characteristic in response to acute tension changes, we used osmotic shock to induce acute tension changes in RPE cells. When imaged by TIRF microscopy, epsin puncta appear to be brighter under hypo-osmotic conditions (Fig. 2d). Cells imaged had similar expression levels of fluorescent proteins (epsin EGFP and mCherry CLC) between different osmotic conditions (Supplementary Fig. 3e). The intensity traces of CCSs belonging to the 60–78 s lifetime cohort showed an increase in the intensity of epsin EGFP when the osmolarity of the media was reduced (Fig. 2e). The average plateau intensity of epsin in other lifetime cohorts (from 10–18 to 100–120 s) was also higher with a decrease in media osmolarity (Fig. 2f), a result similar to what we have observed from the resting cell tension experiments. Our osmotic shock experiments suggest an increase in recruitment of epsin accompanies an acute increase in tension. The intensity of mCherry CLC in CCSs in epsin-expressing cells, belonging to the 60–78 s lifetime cohort, showed a small increase with the acute increase in tension (Supplementary Fig. 3f). CLC plateau intensity in epsin EGFP-recruited CCSs was the same between hyper- and iso-osmotic conditions but significantly increased for hypo-osmotic conditions (Supplementary Fig. 3g). Further, the ratio of maximum intensity of epsin and clathrin was smaller for hyper-osmotic conditions but remained in a comparable range for iso- and hypo-osmotic conditions (Supplementary Fig. 3h). This result is different from the resting cell tension experiments where we observed reduced clathrin intensity, suggesting an acute tension increase has a different effect on clathrin assembly. The intensity profile of epsin starts at a maximum (Fig. 3e), pointing to a delay in the recruitment of clathrin which was used as the primary detection channel in these analyses. To capture the initial intensity profile of epsin, we used epsin as the primary detection channel (Supplementary Fig. 3i), this intensity profile also recaptures the trend we have seen when epsin was a secondary channel (Fig. 3e). Nevertheless, clathrin recruitment into CCSs in cells without epsin overexpression (Supplementary Fig. 4a) was the same when cells were subjected to an acute increase in tension (Supplementary Fig. 4b). However, overexpression of epsin rescues the recruitment of clathrin in CME cohorts at high tension, as shown earlier. This points to the role of epsin in promoting clathrin recruitment when membrane tension increases acutely.

Further, we repeated the osmotic shock experiments in RPE cells overexpressing epsin EGFP after disrupting the actin cytoskeleton using Latrunculin A. As shown previously^12^, an increase in tension together with disruption of actin led to significant stalling of CCSs (Supplementary Fig. 5a–d). The intensity of epsin increases for both iso- and hypo-osmotic conditions when the actin cytoskeleton was disrupted (Supplementary Fig. 5e). This behavior was not seen installed CCSs, suggesting epsin may rescue the internalization of CCSs under high tension in the absence of actin.

### ENTH domain-containing H_0_ helix supports tension-mediated nucleation of epsin at CCS sites

The tension response by epsin to changes in resting tension or acute tension variation is unique from other membrane-associated proteins. Typically, elevated tension reduces the recruitment of proteins to the membrane^30,43^. Paradoxically, epsin shows an increase of recruitment in response to elevated tension. Since the H_0_ helix in the N-terminus of epsin can insert into the bilayer upon membrane binding^16^, we hypothesize that this helical insertion contributes to the unique tension response of epsin. To test this hypothesis, we first removed the ENTH domain of epsin, which is the structured N-terminus region of epsin that contains multiple alpha-helices, including the H_0_ helix. The removal of the ENTH domain of epsin did not render it cytosolic (Fig. 3a), suggesting other parts of epsin can still target it to CCSs. However, it appeared to decrease the number of initiation events of CCSs containing epsin without ENTH compared to full-length epsin (Fig. 3b). Further, as tension increased, overexpressing full-length epsin led to an increase in CCS initiation density (Fig. 3b). This result implies that epsin can sense an increase in membrane tension and respond to it dynamically by increasing CCS nucleation. In contrast, the initiation density of epsin ΔENTH puncta remained unchanged with an increase in membrane tension (Fig. 3b). This points to the ENTH domain playing a role in tension sensitivity of epsin and mediating epsin membrane binding at high membrane tension.

To further delineate the effect of the ENTH domain on tension-mediated recruitment of epsin, we analyzed the intensity of epsin ΔENTH in CCSs in cells co-expressing epsin ΔENTH EGFP and mCherry CLC (Fig. 3c). The average plateau intensity of epsin ΔENTH EGFP in CCPs across multiple lifetime cohorts slightly reduced or remain the same in response to osmotic shock-induced membrane tension increase (Fig. 3d). Removal of ENTH domain abrogated the elevated epsin recruitment to CCSs at high tension. However, the average plateau intensity of clathrin in CCSs with epsin ΔENTH EGFP reduced or remained comparable between iso-osmotic and hypo-osmotic conditions (Supplementary Fig. 6a). This hinted at the possibility that the ENTH domain may be dispensable for epsin-mediated stabilization of clathrin in CCSs.

To understand how the removal of ENTH renders epsin unable to sense tension, We next replaced the residues in ENTH which form the H_0_ helix upon binding PIP_2_^44^ to histidine. These mutations prevent the formation of H_0_ helix when epsin binds to lipid bilayer and blocks amphipathic helix insertion. Dual-color TIRF imaging (Fig. 3e) and downstream analysis of CCPs with mut-H_0_ epsin co-localization show the loss in epsin mut-H_0_’s ability to increase recruitment across different lifetime cohorts from an acute tension increase (Fig. 3f), whereas full-length epsin increases in recruitment as membrane tension increases. This confirmed that the H_0_ helix imparts the tension sensitivity to epsin. This is an important result, as we showed that the ENTH domain with amphipathic insertion can sense membrane tension in addition to the already known ability to detect membrane curvature^44,45^ and dynamically alter the recruitment pattern of epsin to CCSs. The average plateau intensity of clathrin in CCPs with epsin mut-H_0_ EGFP increased slightly or remained in the comparable range as membrane tension increased acutely (Supplementary Fig. 6b). This suggests that epsin-mediated stabilization of clathrin in CCPs can occur without amphipathic insertion of H_0_ into the bilayer. Further, we mutated the PIP_2_ binding site in H_0_ helix RRQMK to SSQMS such that PIP_2_ binding is not possible (Supplementary Fig. 6c). This mutant also showed a similar recruitment behavior as that of epsin ΔENTH and epsin mut-H_0_ (Supplementary Fig. 6d, e). Epsin mut-PIP_2_ also lost the ability to recruit to CCSs with increased membrane tension (Supplementary Fig. 6e). We postulate that PIP_2_ initiates the coiling of the H_0_ helix and the H_0_ senses the changes in membrane tension by detecting changes in the area per lipid. Finally, we reason that endocytic binding domains in the C-terminus region of epsin help its binding to CCSs in the absence of ENTH domain or amphipathic insertion. Based on this assumption, we propose a model for epsin binding for full-length (Fig. 3g (i)), ΔENTH (Fig. 3g (ii)), mut-H_0_ (Fig. 3g (iii)).

### Atomistic insights into ENTH-membrane interactions

**Fig. 4:**
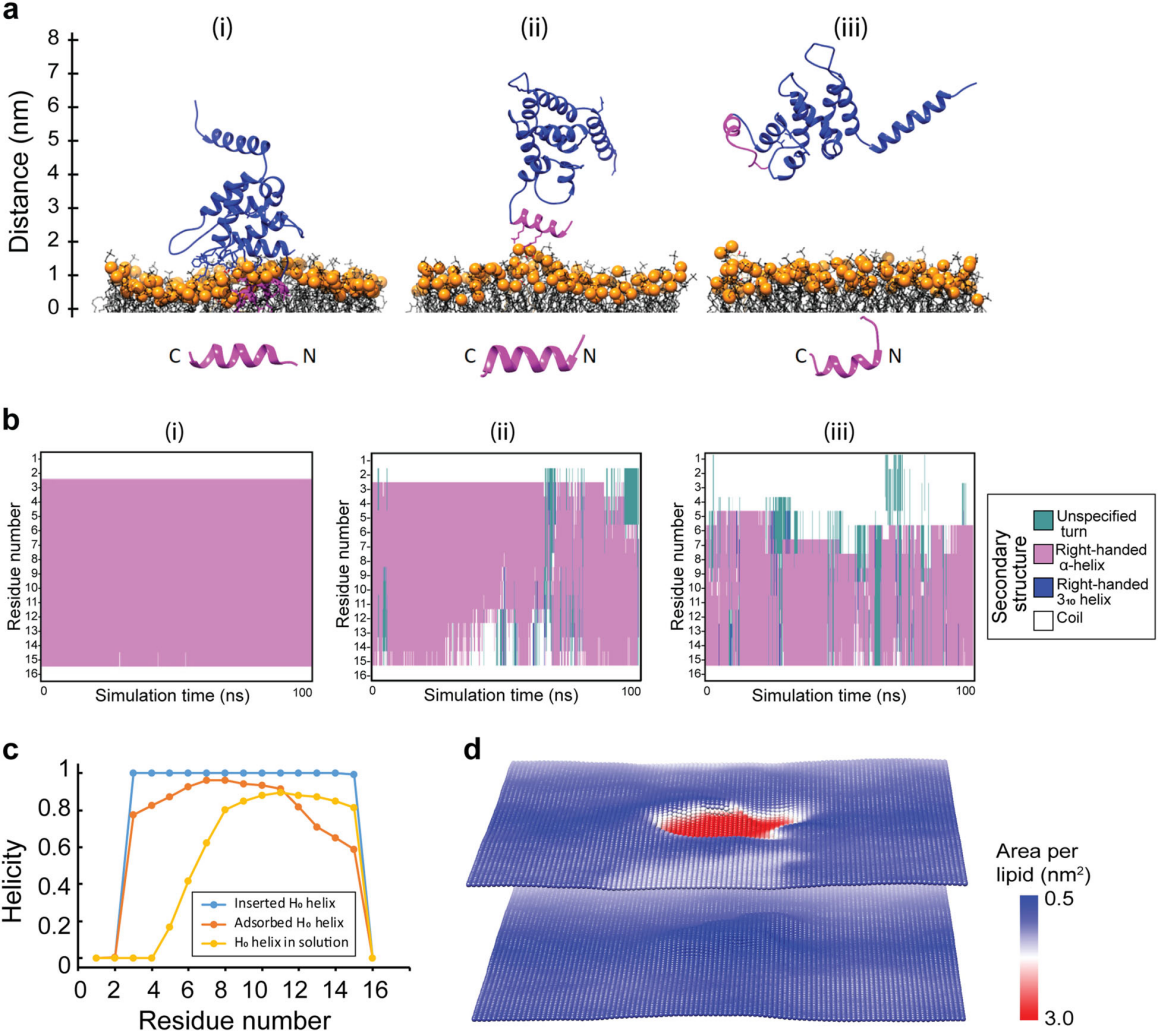
Atomistic insights into ENTH-membrane interactions. **a** ENTH domain interacting with POPC membrane with a single PIP_2_ lipid and pulled out of the membrane. The H_0_ helix shown in magenta is in the inserted (i), adsorbed (ii) and solvent (iii) states. **b** Secondary structure analysis of each residue of H_0_ in the three states shown in (**a**). **c** The summary of the analysis in (**b**). The helicity of H_0_ decreases as it undergoes reduced interactions with the membrane. The protein–lipid interactions stabilize the alpha-helix structure of H_0_. **d** The area per lipid plot corresponding to the inserted state shown in (**a**). The POPC lipids have a typical area of 0.64 nm^2^. The presence of H_0_ displaces lipids in the top leaflet. As a result, the effective area per lipid increases in the H_0_-occupied domain (red region).

To substantiate our intuition, we performed MD simulations to quantify the interactions of the ENTH domain with a lipid membrane made of 1-palmitoyl-oleoyl-sn-glycerophosphocholine (POPC) lipids and a single PIP_2_ lipid using CHARM-GUI. The ENTH domain was placed onto the membrane. This led to an instantaneous interaction of the H_0_ helix with PIP_2_ and the subsequent insertion of the H_0_ helix into the membrane. The ENTH domain was then pulled away from the protein. As a consequence, the inserted H_0_ helix first transitioned to an adsorbed state, and on further pulling was removed from the membrane into the solution. The aforementioned three stages of ENTH-membrane interaction are shown in Fig. 4a. It is notable that the secondary structure of the H_0_ helix undergoes a transformation based on the degree of lipid interactions (Fig. 4a bottom panel). Figure 4b shows the secondary structure analysis as a function of the three stages of the H_0_ helix. In the inserted state, the H_0_ has an alpha-helix structure (pink color). In the adsorbed state with reduced lipid interactions, the H_0_ begins to become disordered from the N-terminus (teal color). In the final state when H_0_ is in the solution, the alpha-helix domain shrinks further transforming into the disordered domain (teal color). The extent of helicity of H_0_ helix residues in the three states is summarized in Fig. 4c.

Figure 4d shows the areal footprint of the H_0_ helix inside the membrane. The plot shows the area per lipid in the two leaflets of the membrane. The POPC lipid area is around 0.64 nm^2^ (blue color). Because of the H_0_ helix insertion, the lipids are moved out of the H_0_-occupied domain. This displacement of lipids effectively increases lipid–lipid separation, which in turn results in an increase in the area per lipid (red color). Since the protein sits primarily in the top leaflet, the change in area per lipid is minimal in the bottom leaflet. This areal footprint plot suggests a potential mechanism for tension sensitivity exhibited by epsin. A single H_0_ helix occupies an area of 2 nm^2^ (red region) and displaces lipids in the membrane. If the membrane has zero resting tension and the lipids are allowed to move freely, there would be no energetic advantage to displacing the lipids. However, if the membrane has a nonzero resting tension (*σ*), the displacement of lipids would be associated with an energetic incentive of −*σ*Δ*A*, where Δ*A* is the area occupied by the H_0_ helix. This idea is similar to the notion that explains the tension sensitivity of mechanosensitive channels in bacterial membranes^46,47^.

### Interfering the formation of H_0_ helix inhibits early recruitment of epsin to CCPs

**Fig. 5:**
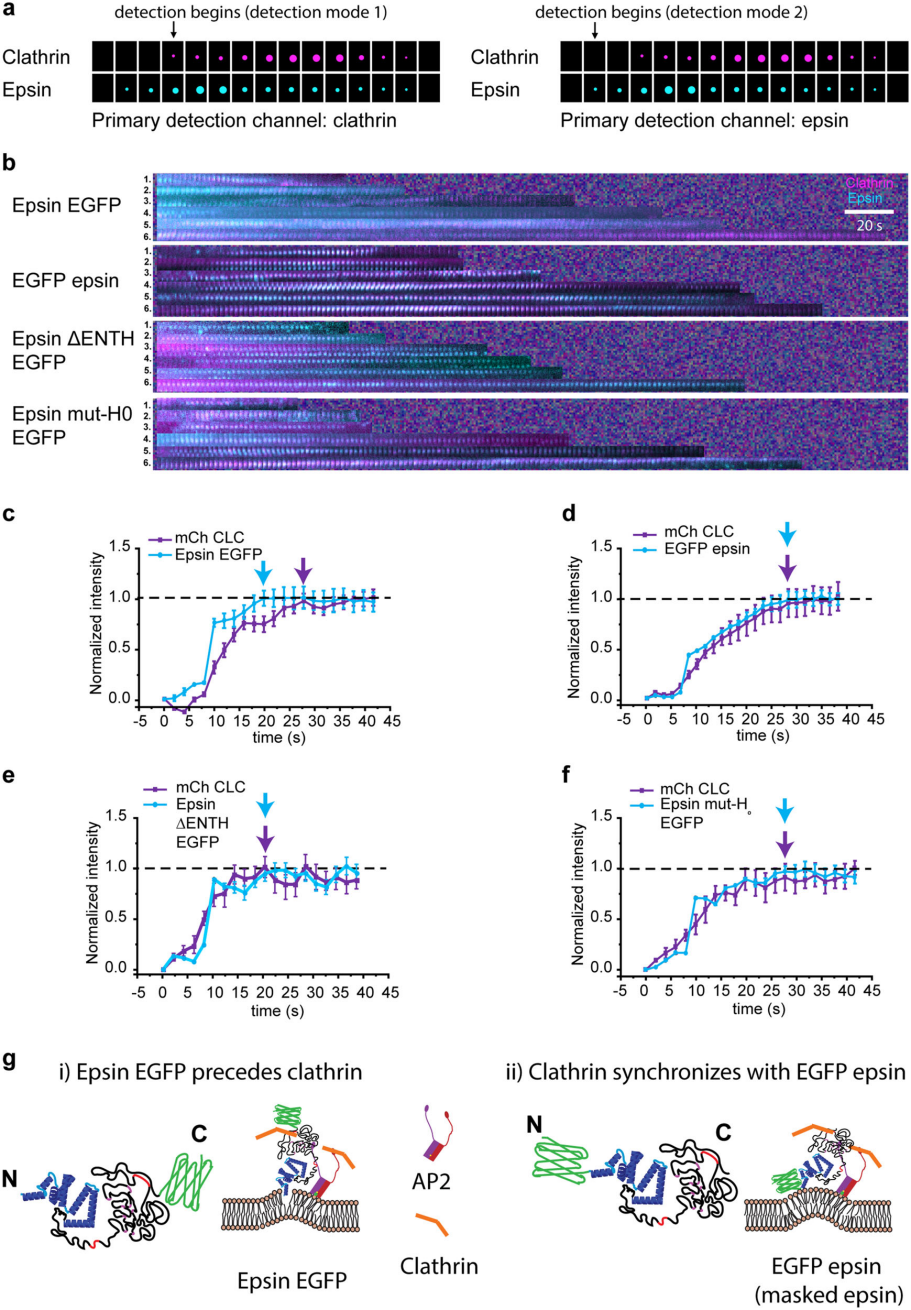
Epsin is recruited prior to clathrin to CCS nucleation sites by amphipathic helix insertion. **a** Schematic of two dual-channel detection modes depicting a scenario in which epsin (cyan) appears prior to clathrin (magenta). **b** Kymographs of CCSs with epsin EGFP, EGFP epsin, epsin ΔENTH EGFP, and epsin mut-H_0_ EGFP (primary detection channel: clathrin). Normalized recruitment of epsin (cyan) and clathrin (magenta) (primary detection channel: epsin) for the 60–78 s lifetime cohorts until they reach maximum intensity for **c** epsin EGFP, **d** EGFP epsin, **e** epsin ΔENTH EGFP, and **f** epsin mut-H_0_ EGFP. **g** Proposed model for epsin recruitment with (i) EGFP in C-terminus and (ii) EGFP in N-terminus. N_cells_ for (**d**–**g**) were 9 (N_tracks_ = 34,777), 7 (N_tracks_ = 56,492), 9 (N_tracks_ = 45,754), and 7 (N_tracks_ = 36,016), respectively. The error bars denote standard error.

The earlier observation that the recruitment of epsin EGFP prior to the arrival of clathrin and the importance of H_0_ helix insertion prompted us to ask whether masking the ENTH domain might alter epsin recruitment to CCPs. Here, we considered epsin with EGFP tagged to the C-terminus and EGFP tagged on the N-terminus (masking the ENTH domain) (Supplementary Fig. 7a), in addition to epsin ΔENTH EGFP and epsin mut-H_0_ EGFP mutants, and examined kymographs of different epsin mutants. Using CCP tracks with detection that started at the first significant clathrin signal (Fig. 5a, detection mode 1), we determined the order of arrival of epsin with respect to clathrin. Epsin with EGFP attached to the C-terminus arrived at CCPs prior to clathrin, as the kymograph showed more green fluorescence at the beginning of the lifetime track (Fig. 5b panel 1), pointing to the early recruitment of epsin prior to clathrin and consistent with what we observed in Fig. 2b, f. In contrast, the masked ENTH showed synchronous recruitment with clathrin (Fig. 5b panel 2). This finding is consistent with the work of Taylor et al.^48^, which showed delayed recruitment of epsin compared to AP2 when fluorescent protein is placed in the N-terminus of epsin. Similarly, both epsin ΔENTH EGFP and epsin mut-H_0_ EGFP helix mutants showed synchronous recruitment with clathrin (Fig. 5b panels 3 and 4). These data were further quantified by plotting the normalized intensities of clathrin and epsin until they reached their maximum intensity after performing CCP detection and tracking with epsin as a primary channel (Fig. 5a, detection mode 2). The normalized epsin EGFP recruitment curve was above the mCherry-clathrin recruitment curve, pointing to the early recruitment of epsin (Fig. 5c). In addition, epsin EGFP reached maximum recruitment prior to mCherry CLC (shown with cyan and magenta arrows). However, cells expressing epsin with masked or deleted ENTH domain or with mutated H_0_ helix showed epsin recruitment in synchrony with mCherry CLC (Fig. 5d–f). Based on these results, we suggest that epsin EGFP recruitment to membrane precedes clathrin (Fig. 5g case i), whereas masking ENTH domain with EGFP disrupts or delays the formation of H_0_ amphipathic helix insertion into the bilayer (Fig. 5g case ii). This is also supported by the synchronous recruitment of epsin mut-H_0_ EGFP and epsin ΔENTH with clathrin, as they cannot form H_0_ helix upon binding to the membrane.

### Bi-directional stabilization of epsin recruitment to CCSs and curvature of CCS domes is mediated by the unstructured domain of epsin

**Fig. 6:**
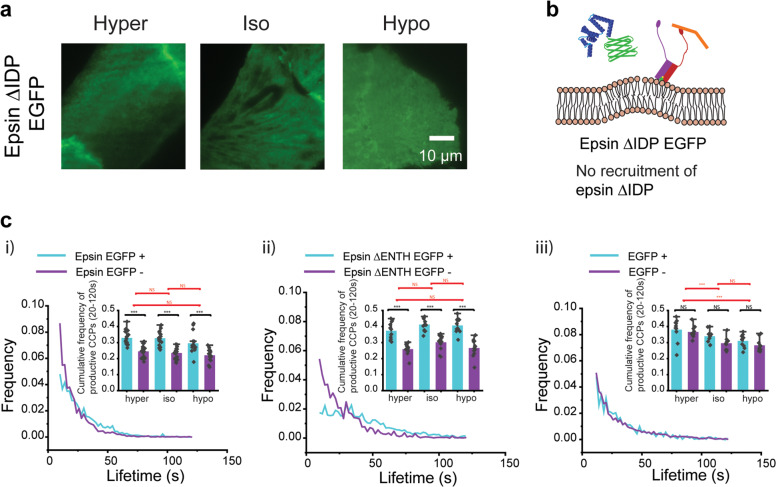
Bi-directional stabilization of epsin recruitment in CCSs and curvature of CCS domes to form productive CCSs is mediated by unstructured IDP region. **a** Fluorescence images of epsin ΔIDP under different tension conditions (hyper-, iso-, and hypo-osmotic conditions). **b** Proposed model of interaction between a CCS and epsin ΔIDP. **c** The lifetime distribution of CCSs with (cyan) or without (magenta) epsin EGFP for an iso-osmotic condition for (i) full-length epsin with C-terminus EGFP, (ii) epsin ΔENTH EGFP, and (iii) EGFP (control). Inset shows cumulative frequencies of mature CCSs with lifetimes 20–120 s for the different osmotic conditions indicated. The N_cells_ expressing epsin EGFP for hyper-, iso-, and hypo-osmotic conditions in (**c**) were 19 (N_tracks_ = 38,282), 19 (N_tracks_ = 58,574), and 16 (N_tracks_ = 32,644), respectively. The N_cells_ expressing ΔENTH EGFP epsin for hyper-, iso-, and hypo-osmotic conditions in (**c**) were 12 (N_tracks_ = 19,328), 12 (N_tracks_ = 20,078), and 12 (N_tracks_ = 31,952), respectively. The N_cells_ expressing EGFP for hyper-, iso-, and hypo-osmotic conditions in (**c**) were 12 (N_tracks_ = 23,670), 14 (N_tracks_ = 24,615), and 13 (N_tracks_ = 23,424), respectively. The error bars denote standard error. NS denotes not significant. *, **, *** represent *p* < 0.05, *p* < 0.01, and *p* < 0.001, respectively.

Removal of the ENTH domain did not render epsin cytosolic. Hence, to determine which domains are responsible for the stabilization effect of epsin, we progressively removed binding domains from epsin within the unstructured IDP region (Supplementary Fig. 7b). Removal of clathrin binding domain (CBD) 1 (LMDLADV) or CBD2 (LVDLD) did not affect epsin recruitment into CCSs (Supplementary Fig. 8 (i) and (ii))^49^. Simultaneous removal of CBD1 and CBD2 also did not inhibit the formation of epsin puncta (Supplementary Fig. 8 (iii)), nor was the number of epsin puncta in these mutants affected by an acute increase in membrane tension via osmotic shock (Supplementary Fig. 8 (i)–(iii), top and bottom panel). However, removal of DPW repeat motifs, which bind to AP2 subunit along with CBD1 and CBD2, renders epsin cytosolic (Supplementary Fig. 8 (iv)). Using a transferrin-uptake assay, we showed that overexpression of epsin mutants did not affect cargo recruitment and internalization via CME (Supplementary Fig. 9), suggesting that mutant epsins did not impact CME. Consistent with earlier findings, removing the unstructured IDP region of epsin containing all the endocytic binding sites also resulted in epsin being cytosolic, and this is true under all tension conditions (Fig. 6a). This implies that ENTH, which is the structured domain of epsin containing H_0_ helix, alone cannot stabilize epsin recruitment to CCSs, but it requires the AP2 and clathrin binding sites in the C-terminus unstructured region of the protein. This is an important result as we have shown that the ENTH domain mediates tension-dependent nucleation of epsin and in vitro studies have shown ENTH domain alone is recruited to pre-curved lipid bilayers containing PIP_2_^16,45^. To further confirm the requirement of AP2 and clathrin binding to stabilize epsin recruitment to the plasma membrane, we used shRNA knockdown of clathrin heavy chain (CHC) or α-adaptin in the AP2 subunit. Knocking down CHC or α-adaptin that blocks the formation of CCSs also inhibited the formation of epsin puncta (Supplementary Fig. 10). This behavior was observed in full-length epsin, epsin ΔENTH, and epsin ΔIDP expressing cells. This provides support that binding of epsin to CHC and AP2 is necessary for stable recruitment of epsin and the ENTH domain alone cannot support plasma membrane recruitment (Fig. 6b).

Productive CCPs (lifetime 20–120 s) internalize into cytoplasm without prematurely dissembling or stalling on the membrane. Our SIM-TIRF data showed that overexpression of epsin supports the maturation of CCSs to productive CCPs. Furthermore, dual-color SIM-TIRF data showed that the presence of epsin tagged with EGFP preferentially supports the formation of productive CCPs (shown by the preferential formation of rings when epsin is present (green) in Fig. 1c), suggesting that epsin may stabilize productive CCPs. To determine the effect of the epsin recruitment on the stability of CCSs, we classified CCS tracks as with or without co-localization with epsin tagged with EGFP. We found that the fraction of productive CCPs in a cell is higher in epsin-containing CCPs (Fig. 6c (i)). We also found that there was no statistically significant change in the fraction of productive CCPs between hyper-, iso-, and hypo-osmotic-treated cells (Fig. 6c (i) inset). Similarly, cells having CCSs with epsin ΔENTH recruitment also showed an increased fraction of productive CCPs (Fig. 6d (ii)), again independent of membrane tension. As a control, we looked at whether cells expressing EGFP had the same fraction of productive CCPs in different tension conditions. For this analysis, we used the false positive puncta detection from the secondary channel (EGFP) to classify pits as EGFP-positive (EGFP+) and EGFP-negative (EGFP−). Comparison of the fractions of productive CCPs which are EGFP+ and EGFP− showed no difference between them (Fig. 6d (iii)), unlike epsin-positive CCSs in epsin or epsin ΔENTH expressing cells which showed significant increases in the population of productive CCPs. As tension increased, the fraction of productive CCPs decreased for cells expressing only EGFP, consistent with the previous finding from our lab^30^. Altogether, these findings show that epsin supports the formation of productive CCPs. With the help of endocytic binding sites in the IDP domain that confers steric crowding, epsin is able to stabilize the curvature of CCS domes. ENTH domain, however, does not play an active role in providing this stabilization effect. Together, these results demonstrate a bi-directional stabilization of epsin recruitment by binding to AP2 and CHC and stabilization of productive CCPs due to binding of epsin to AP2 and CHC, both mediated by the unstructured IDP region of epsin.

## Discussion

**Fig. 7:**
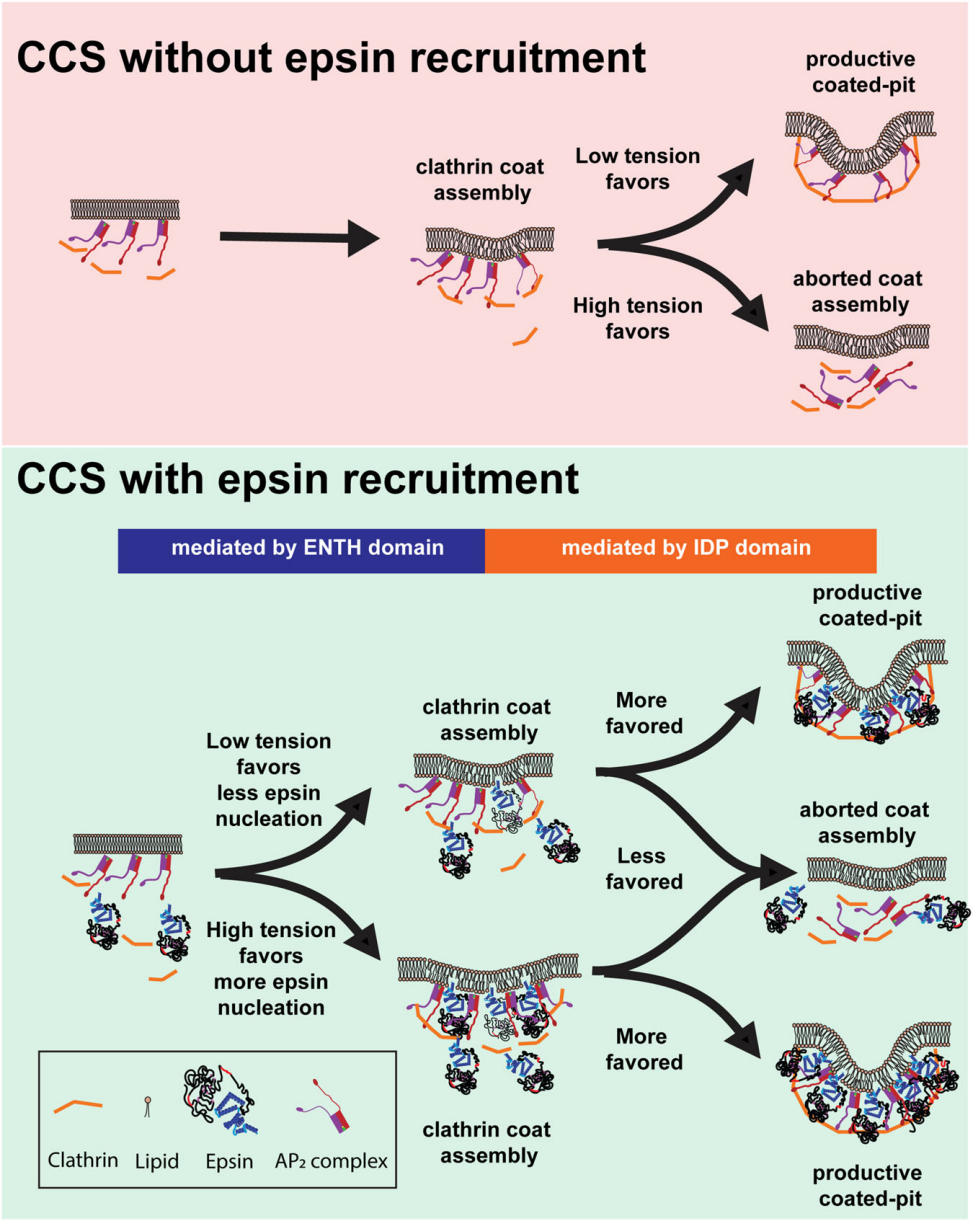
Summary of the role of epsin in CCS formation under different tension environments. Epsin provides enhanced CCS stability compared to CCSs without epsin. At high tension, more epsin molecules are recruited to counteract elevated tension and to provide stability to CCSs. The AP2 and clathrin binding of epsin primarily impart the additional stability to coated pits by anchoring constituents of the CCS complex together.

Membrane tension is known to be a regulating factor of different endocytic pathways^12–14,43^. Prior experimental evidence points to actin dynamics in supporting the transition of CCSs from hemispherical domes to omega-shaped pits during CME at high tension^12^. However, it has been puzzling how cells overcome the transition from the flat membrane to hemispherical domes during CME at high tension. The exact mechanism of curvature formation in CCPs is hotly debated^8,38,50,51^ and whether membrane tension and presence of membrane bending proteins during CCP initiation control fate of the CCP is an open question. Here we uncovered epsin’s ENTH and IDP domains play complementary roles to ensure the completion of CCS maturation under high tension environments (Fig. 7). Under a SIM-TIRF field, a productive CCS track shows the evolution of the hemispherical dome manifested as a ring^38^. We found that overexpressing epsin or epsin EGFP in cells supports the maturation of coated pits at high tension. Utilizing dual-color TIRF imaging, we showed that epsin EGFP recruitment into CCSs increases with an increase in resting membrane tension or acute membrane tension. We showed that masking ENTH domain activity in epsin (i.e., by placing EGFP at the N-terminus end of epsin) blocks the early recruitment of epsin to the CCS sites. The IDP domain provides binding to AP2 and clathrin, which is required for epsin’s ability to stabilize CCSs since ENTH domain alone is cytosolic. Our finding is important as most membrane-associated proteins disassemble or dissociate from the lipid bilayer as membrane tension increases^30,43,52^. The complementary actions of ENTH and the unstructured IDP region of epsin enable it to detect membrane tension variations to support the flat-to-dome transition in high-tension environments. Interestingly, we find that epsin is recruited early before clathrin, in contrast to what Taylor et al. reported^48^. This is due to the fact that Taylor et al. used an N-terminus epsin fusion protein which blocks ENTH function. Thus, our findings would be consistent with epsin’s role in tension sensing.

Previously, Brady et al.^53^ showed that both ENTH and C-terminal domain of epsin regulate its dynamic interaction with CCSs in *Dictyostelium*. Similarly, work in yeast from the Wendland lab has shown that the ENTH domain of epsin is essential for endocytosis by binding to phosphoinositides and other endocytic constituents like clathrin as well as playing a role in downstream signaling through the Cdc42 signaling pathway^54–56^. In contrast, our experiments showed that the C-terminus IDP domain that contains AP2 and clathrin binding sites is sufficient for epsin to target CCSs. Further, Zeno et al.^45^ found that disordered domains enhance the curvature sensitivity of structured domains^45^. Similarly, we found that the synergy between a structured (i.e., ENTH) domain and an unstructured (i.e., IDP) domain can achieve membrane tension sensing.

The increase of epsin recruitment at high tension is dependent on the ENTH domain, and specifically the H_0_ helix. Our observation that undisrupted activity of ENTH, specifically the amphipathic insertion of the H_0_ helix, is required for early epsin recruitment would be consistent with its tension-sensing role. Importantly, it is consistent with a previous finding where epsin recruits clathrin to PIP_2_-containing lipid monolayer and stimulates clathrin lattice assembly^16^. When ENTH of epsin is masked by N-terminal EGFP, its recruitment to CCS sites synchronizes with clathrin, presumably by binding to AP2 via epsin’s IDP domain. This is in agreement with our finding that removing the ENTH domain did not inhibit the recruitment of epsin to CCSs but only affected its early recruitment. Because the unstructured IDP region contains multiple clathrin and AP2-binding sites, it is likely that the stability of CCSs at high tension is afforded by the avidity provided by multiple interactions^7^. The importance of multiple interactions is reinforced by the finding that knocking down either CHC or α-adaptin renders full-length epsin cytosolic. Although epsin ΔENTH can be recruited to sites of CCSs, this recruitment is reduced drastically when tension is increased, similar to the behaviors of other membrane-associated proteins when encountering high tension.

Using MD simulation, we showed that insertion of the H_0_ helix into the lipid bilayer enables the recruitment of ENTH into the membrane. The simulations also show that membrane-association triggers the ordering of H_0_ into a helical structure. Because of the favorable H_0_–lipid interactions, ENTH prefers to bind to the membrane. Also, the H_0_ helix insertion displaces lipids, thereby providing an energetic advantage for epsin recruitment in a high membrane tension regime. Tension-induced amphipathic helix insertion could perhaps be a general mechanism for membrane tension sensitivity for other cellular machinery. Any protein with a membrane-embedded segment could be expected to demonstrate tension sensitivity. However, the propensity to sense tension and undergo recruitment would likely be proportional to the areal footprint of the inserted protein segment. This could be a prime reason that allows an amphipathic helix to act as a tension sensor as it lays tangential to the membrane, displacing a higher number of lipids in contrast to an orientation normal to the membrane. Another potential example of this mechanism could be occurring in nucleation promoting factor N-WASP. Under load, actin networks become denser to support the increased load^57^. In this context, increased membrane tension should therefore promote actin assembly, and this can be achieved by increasing actin filament nucleation. Interestingly N-WASP has an amphipathic helix^58^. Thus, it is plausible, although highly speculative, that recruitment of N-WASP to the plasma membrane could be tension-sensitive in a fashion similar to epsin.

The ability of the H_0_ helix to detect curvature versus to generate curvature is an ongoing debate. It is already shown that the H_0_ helix can preferentially recruit to areas of high curvature^26,44,59^. Our MD simulations show that the area per lipid molecule increases as membrane tension increases. Area per lipid values also increase as membrane curvature increases. Membrane scaffolding proteins like BAR proteins and epsin have been shown to recruit selectively to highly curved membrane tethers kept at high tension^26,60^. An increase in tension in this finite membrane tether reduces the tether radius and thereby increasing curvature. Similarly, an increase in area per lipid due to an increase in tension may lead to recruitment of epsin which facilitates membrane bending. Initial membrane bending by nucleating epsin can further increase the local area per lipid around nucleation sites further increasing epsin recruitment. This will lead to a positive feedback loop of membrane curvature sensing and membrane curvature generation.

Although the H_0_ helix can insert into the membrane and acts as the tension sensor, our data does not support that ENTH alone as the curvature generation machinery. The ability of the unstructured IDP region to tether different endocytic proteins (AP2, clathrin), besides generating steric pressure, is partly responsible for the stabilization effect of CCSs under high tension (i.e., epsin-positive CCSs have a larger fraction of productive structures). We speculate that steric repulsion by the bulky unstructured IDP region also plays a role in inducing and stabilizing the curvature of the dome structure^22,23^. While stable epsin recruitment to CCSs requires the presence of CHC and AP2, once epsin is recruited to CCSs it supports the formation of the CCS dome with the help of its unstructured IDP region. Interestingly, cells overexpressing epsin EGFP have more productive/stalled CCSs under high tension compared to cells overexpressing epsin. The EGFP following the unstructured IDP region increases its bulkiness and therefore steric pressure. Our work implies the existence of a bi-directional stabilization between endocytic components of CCSs and IDP-mediated curvature generation.

Elevated membrane tension plays an inhibitory role in CME^10,11^. We believe cargo uptake via CME is dependent on the total number of productive CCPs on the cell membrane. Transferrin uptake in cells reduced as membrane tension increased in RPE cells with endogenous level epsin. Our SIM-TIRF data showed that increase in membrane tension reduced the number of productive CCPs in RPE cells. However, the number of productive CCPs did not reduce when epsin was overexpressed. This result is in good agreement with our transferrin-uptake assay which showed similar rate of uptake for cells overexpressing epsin under different membrane tension conditions. This reinforces our finding that epsin plays an integral role in membrane curvature stabilization under high tension which is necessary for the formation of productive CCPs.

Although we have identified a new mode of epsin function, we speculate epsin is not unique in mediating tension-responsive recruitment to and stabilization of CCSs. In particular, adapter protein 180 (AP180) and its homolog clathrin assembly lymphoid myeloid leukemia protein (CALM) both have alpha-helices in their N-terminal domains^61^ along with an unstructured IDP region consisting of endocytic binding domains^18^. Thus, it is plausible that a similar mechanism could be at play with AP180 and CALM to support CME under high-tension environments. Several works using MD simulation and in vitro experiments have shown the role of amphipathic helices, specifically H_0_ helix, in curvature generation mechanism of N-BAR proteins^27,44,59,62^. It should be investigated whether H_0_ helix insertion of these proteins also enables tension-mediated membrane curvature generation and stabilization. Our work also provides some unexpected findings for future investigation. While we mainly focused on the role of epsin in high-tension environments, decreasing tension in hypertonic solutions resulted in distinct responses. In particular, the steady-state density of epsin ΔENTH puncta under a hyper-osmotic condition was significantly higher compared to the iso-osmotic condition. Furthermore, recruitment of epsin ΔENTH or epsin mut-H_0_ was significantly elevated under hyper-osmotic conditions. Interestingly, there appeared to be more clusters of CCSs in our SIM-TIRF images under hyper-osmotic conditions. It has been suggested recently that lowering membrane tension increases caveolar cluster formation^63^. Although clusters of CCSs have been postulated to be non-terminal endocytic events^64^, it will be intriguing to see if these represent local hot spots where membrane tension is low. There exist multiple endocytic proteins like AP2 and FCHo that perform the same function of membrane curvature generation and stabilization in CME^8,65,66^. Further, epsin is not expressed at elevated levels in all cell types. There were also a significant proportion of CCPs which did not have epsin recruitment. Hence, more research should be done in exploring the presence of these redundant membrane sculpting mechanisms in CME. It will also be interesting to know how the arrival time of different adapter proteins may regulate their membrane bending capabilities. It may be the case that different membrane-binding proteins may facilitate curvature generation and stabilization in a spatiotemporally regulated manner^5,14,48^.

Finally, how might epsin’s recruitment to CCSs under high tension be important to cell physiology? Changes in cell tension is expected to trigger various cell signaling responses. Epsin has a ubiquitin-interacting motif with the dual function of binding ubiquitin and promoting ubiquitylation. Thus, regulating epsin recruitment to CCSs has important implication of ubiquitinated cargo endocytosis^67^ and signaling. The association of epsin with ubiquitinated cargo is negatively regulated by clathrin^68^. An increase in resting cell tension increases epsin recruitment and decreases clathrin recruitment. Coupled with the increased stability of epsin-positive CCSs, our findings would suggest that high membrane tension would impact ubiquitinated cargo endocytosis and signaling. The intersection of mechanosensing and endocytic regulation remains an under-studied area that will have profound implications in mechanotransduction.

## Methods

### Cell culture

RPE cell was a gift from Sandra Schmid (UT Southwestern Medical Centre). The cells were maintained in Dulbecco’s Modified Eagle Medium with nutrient mixture F-12 (DMEM/F-12) supplemented with 10% (v/v) fetal bovine serum (FBS) and 2.5% (v/v) penicillin/streptomycin at 37 °C and 5% CO_2_.

### Generation of epsin EGFP and EGFP epsin lentiviral constructs

All constructs generated for this work have been sequenced for accuracy. EGFP epsin from pMIEG3 vector (gift from Sandra Schmid, original construct from JoAnn Trejo, UCSD) was amplified with the following primers (Integrated DNA Technologies Inc.) and cloned into pLVX puro vector, using BsrGI and Xbal digestion: Forward primer—5′-ATAATATGTACAAGTCCGGACTCAGATCTC-3′; Reverse primer—5′-GCGGCGTCTAGATTATAGGAGGAAGGGGTT-3′. Epsin is amplified from pLVX puro vector, using the following primers and cloned into pLVX EGFP puro vector, using XhoI and EcoRI digestion to create epsin EGFP construct: Forward primer 5’ – CATCATCTCGAGGCCACCATGTCGACATCATCGCTG-3′; Reverse primer-5′-ATAATAGAATTCTCCACTTCCACTTCCACTTCCTAGGAGGAAGGGGTT-3′.

Both constructs were transformed into competent *E. coli* cells (New England Biolabs Inc.) and selected using antibiotics (Ampicillin).

### Generation of epsin mutant constructs

Epsin mutant constructs were generated successively by mutagenesis using Q5® Site-Directed Mutagenesis Kit (New England Biolabs Inc.). CBD1 was deleted using following primers to create epsin ΔCBD1 EGFP: Forward primer 5′- TTCACAACCCCAGCCCCT-3′; Reverse primer 5′-AGATGACTCCTCCTTGCCC-3′. CBD2 is deleted from epsin EGFP and epsin ΔCBD1 EGFP using following primers to create epsin ΔCBD2 EGFP and epsin ΔCBD1 and 2 EGFP: Forward primer 5′-TCACTGGTGAGCCGACCA-3′; Reverse primer 5′-GGCTGCATTAGGGCCTAG-3′. Epsin amino acid sequence containing endocytic binding cites CBD1, CBD2, and AP2-binding motifs were deleted using following primers using to create epsin ΔEBD EGFP: Forward primer 5′-TCACTGGTGAGCCGACCA-3′; Reverse primer 5′-AGATGACTCCTCCTTGCCC-3′. ENTH domain of epsin was removed using the following primers to generate epsin ΔENTH EGFP: Forward primer 5′-GCCCACGCGCTCAAGACC-3′; Reverse primer 5′-CATGGTGGCCTCGAGATCTGAG-3′. Unstructured IDP region of epsin was removed using the following primers to generate epsin ΔIDP EGFP: Forward primer 5′-GGAAGTGGAAGTGGAAGTGGAGAATTCG-3′; Reverse primer 5′-GCGCTCCTCCCGAAGCCG-3′. H_0_ helix in ENTH domain of epsin was substituted to histidine using following primers to generate epsin mut-H_0_ EGFP: Forward primer 5′-CACCACCACAACTACTCAGAGGCAGAGATC-3′; Reverse primer 5′-ATGATGATGTGTCGACATGGTGGCCTC-3′. PIP_2_ binding site in H_0_ helix of ENTH domain was mutated from RRQMK to SSQMS using the following primers to generate epsin mut-PIP_2_ EGFP: Forward primer 5′-GATGAGCAATATCGTCCACAACTACTC-3′; Reverse primer 5′-TGGCTGGACAGCGATGATGTCGACATG-3′. All mutant constructs were transformed into competent *E. coli* cells (New England Biolabs Inc.) and selected using antibiotics (Ampicillin).

### Lentivirus transduction

Lentiviral vectors encoding epsin or epsin mutants were generated by transfecting 70% confluent HEK 293T for 24 h with a plasmid cocktail. The plasmid DNA cocktail contained 1.875 µg psPAX2, 625 ng pMD2.G, 2.5 µg pLVX vector diluted in 3 mL Opti-MEM (Thermo Fisher) containing 15 µL Lipofectamine 2000 (Thermo Fisher) and 5 mL of DMEM media with 10% FBS (v/v). The plasmid cocktail was replaced with fresh DMEM media containing 10% FBS after 24 h. The supernatant was harvested after 48 h, filtered through 0.3 µm sterile filter and flash frozen using liquid nitrogen.

### Stable cell line generation

RPE cells stably expressing EGFP-tagged epsin or epsin mutant and red fluorescent protein (mCherry)-tagged CLC a (mCherry CLC) were generated in a two-step process. RPE cells were transduced with retroviruses (encoding mCherry CLC) in a pMIEG3 vector produced by the UM vector core, followed by FACS sorting (UM Flow Cytometry core) to generate stable RPE cells expressing mCherry CLC. RPE cells expressing mCherry CLC have further infected with lentivirus encoding EGFP epsin or epsin mutants followed by antibiotic selection (Puromycin) to generate double stable RPE cells.

### shRNA knockdown

shRNA constructs for epsin/alpha adaptin/CHC was created in pLKO.1 vector by the UM vector core. Lentiviruses encoding shRNAs of interest were generated using the aforementioned lentivirus transduction method. RPE cells were infected with the viruses and selected using antibiotic selection (Puromycin). All downstream assays (TIRF imaging or Western blotting) were performed on day 5 of transduction and the level of knockdown was confirmed by Western blot analysis.

### Cell spreading on fibronectin islands

Polydimethoxysiloxane (PDMS) stamps with square shapes of size 25 and 32 µm were created from a silicon master mold made by using soft lithography. Sylgard-184 elastomer and curing agents (Dow Corning, Midland, MI) were mixed at a ratio of 10:1 (w/w) and casted over the silicon mold and cured at 60 °C overnight. Fibronectin (Sigma) solution (40 μg/ml) was added onto the stamps and incubated for 1 h at room temperature. The stamps were blown dry using filtered air and placed in conformal contact with UV–ozone-treated PDMS-coated coverslip. Coverslips were spin-coated with a layer of PDMS diluted in hexane (1:20) at 5000 rpm for 2 min. PDMS-coated coverslips enable the efficient transfer of stamped proteins. Immediately after stamping, the coverslip was passivated with 0.1% (v/v) Pluronic-F127 (Sigma) for 1 h, followed by extensive washing with phosphate-buffered saline (PBS). RPE cells were seeded on the coverslips and allowed to selectively adhere to the square patterns. After 1 h of seeding, media was changed to remove non-adhering cells. The adherent cells were allowed to fully spread for 5 h, followed by TIRF imaging.

### Osmotic shock

The RPE cells were imaged in three media having different osmolarities: (i) hyper—440 mmol/kg, (ii) iso—290 mmol/kg, (iii) hypo—220 mmol/kg) were used. Hyper-osmotic solution was prepared by adding 150 mM sucrose to phenol red-free DMEM containing 2.5% FBS. Iso-osmotic solution was DMEM media with 2.5% FBS. The hypo-osmotic solution was prepared by adding deionized water containing 2.5% FBS to DMEM media containing 2.5% FBS in 1:3 ratio. All media osmolarities were checked on a Vapro osmometer. The cells were imaged between 5 and 35 min of adding the different media.

### Actin cytoskeleton disruption

RPE cells overexpressing epsin EGFP and mCherry clathrin were incubated with Latrunculin A (0.5 µM) (Thermo Fisher Scientific) for 30 min prior to TIRF imaging for disrupting actin cytoskeleton.

### Confocal microscopy

The 3D profiles of cells subjected to different osmotic conditions were imaged using Olympus-IX81 microscope with spinning disk confocal scanner unit (CSU-X1; Yokogawa, Japan), EMCCD camera (iXon X3; Andor, South Windsor, CT), 60× objective (NA = 1.42). A z-step size of 0.2 µm was used. EGFP was used as volume marker. The 3D reconstruction was performed using 3D projection plugin in ImageJ.

### Micropipette aspiration

Variation in membrane tension due to osmotic shock was quantified by using micropipette aspiration. Glass micropipettes of inner diameter of ~5 µm were fabricated by pulling borosilicate glass pipette (BF100-50-10; Sutter Instrument) using a micropipette puller (Sutter Instrument). The micropipette was attached to a custom-made stage with pipette holder assembly (MI-10010; Sutter Instrument). An open chamber was made on coverslip seeded with RPE cells using VALAP sealant. The cells were subjected to osmotic shock, using the aforementioned method. The micropipette was made into contact with the cells with the aid of brightfield imaging using an inverted microscope (Nikon TiS) equipped with 20× objective and a CCD camera (CoolSNAP MYO). A negative hydrostatic pressure of 2.156 kPa was applied to the cell to aspirate the plasma membrane of cells into the pipette. The ratio of equilibrium protrusion length of membrane (*L*_*p*_) vs. inner radius of pipette was measured for different osmotic conditions.

### Live-cell imaging via TIRF microscopy

RPE cells expressing the constructs of interest were plated on a coverslip at a low concentration (~1.7 × 10^5^ cells per 22 × 22 coverslip) and allowed to spread for 12–16 h. TIRF microscopy was performed using a Nikon TiE-Perfect Focus System (PFS) microscope equipped with an Apochromat 100× objective (NA 1.49), a sCMOS camera (Flash 4.0; Hamamatsu Photonics, Japan), and a laser launch controlled by an acousto-optic tunable filter. Cells were imaged at 2 s intervals (100 ms exposure) for 5 min at 37  °C with dual-color excitation of 488 and 561 nm lasers (Coherent Sapphire).

### Image analysis for CCS dynamics

Image analysis was performed using CMEAnalysis software developed by Aguet et al.^35^ mCherry-clathrin channel was assigned as the primary detection channel and EGFP as the associated secondary channel. The program uses Gaussian mixture model fitting to detect and localize CCSs. It also performs CCS tracking using µ-track package with a gap-closing feature generating trajectories of CCSs. The intensities of the images were background-corrected along with photo-bleaching correction between time steps. For tracks which are valid (validity of a track as CCP is determined by the gap length), a further thresholding step was performed to remove faint tracks which may not be actual CCSs. Only CCSs with a lifetime between 10 and 120 s were considered for the downstream analysis. The program generates the lifetime and intensity data for CCSs under two categories (i) CCSs containing EGFP epsin and (ii) CCSs not containing EGFP epsin. These were further classified into six cohorts according to their lifetimes (cohorts (10–18, 20–38, 40–58, 60–78, 80–98, and 100–120 s). For quantification of the early arrival of epsin, aforementioned analysis was repeated with EGFP as the primary detection channel and mCherry as the associated secondary channel.

### Live-cell imaging via structural illumination microscopy in TIRF mode (SIM-TIRF)

RPE cells expressing the constructs of interest were plated on a MatTek dish at a concentration of ~1.7 × 10^5^ cells per dish for 12–16 h. SIM-TIRF was performed using a Nikon N-SIM microscope equipped with an Apochromat 100× objective (NA 1.49) and an sCMOS camera (Flash 4.0; Hamamatsu Photonics, Japan). Epsin EGFP was imaged using 488 nm laser with exposure time of 200 ms, with nine images taken in TIRF mode with linear translation of Moire pattern for SIM reconstruction. Similarly, mCherry clathrin was imaged using 561 nm laser with exposure time of 500 ms. The time interval between each set of SIM reconstruction images were 5 s. Dual-color SIM-TIRF images for epsin EGFP and mCherry clathrin was performed using DeltaVision OMX SR system (GE) equipped with 60× 1.42 NA objective and a sCMOS camera. Epsin EGFP and mCherry clathrin were imaged using 488 and 561 nm lasers respectively, with exposure times of 50 ms. Nine images were taken in ring TIRF mode with the angular translation of Moire pattern for SIM reconstruction.

### Image reconstruction and analysis for SIM-TIRF

Images captured in Nikon N-SIM system was reconstructed into SIM images using Nikon Elements software. Reconstructed images were further equalized in intensity across time periods using Nikon Elements software. CCSs were detected and tracked using Trackmate plugin in FIJI (ImageJ)^69^. A detector with Laplacian of Gaussian Filter is applied to detect CCSs with a quadratic fitting scheme for subpixel localization and an estimated blob size parameter of 500 nm. A simple linear assignment problem tracker was applied with a maximum linking and gap-closing distance of 500 nm and maximum gap closing of 2 frames. CCSs were morphologically characterized into abortive, productive, and stalled structures.. Using lifetimes limits for abortive, productive and stalled CCS, the fraction of CCSs belonging to each category was determined.

### MD simulations

The MD simulation systems were composed of a bilayer with an ENTH domain of epsin (PDB number 1H0A^16^), a PIP_2_ molecule, and 99 POPC lipids in the top leaflet and 100 POPC molecules in the bottom leaflet. In addition, the solution consisted of TIP3 (an all atom model of water) water molecules with 0.15 mM KCl. The simulations were performed in GROMACS using 303.15 K and 1 bar using CHARMM36 force field^70^.

The file taken from the protein data bank contained an ENTH domain of epsin and a PIP_2_ head in the inserted configuration. The head group was replaced with the entire lipid preserving the position and original orientation of the head group. Then, the POPC molecules were inserted in a grid pattern in order to construct the bilayer. Finally, the solution with the corresponding KCl concentration was added. All the files detailing the molecule and the equilibration procedure were obtained from CHARMM GUI website^71^.

In order to compute different positions of ENTH with respect to the membrane, we pulled the protein sequentially starting from the inserted configuration by imposing a restraining force on the center of mass of the protein. Additional restraints were applied to the PIP_2_ lipid to prevent it from leaving the membrane. The pulling proceeds until the protein are not in contact with the membrane. All protein configurations were given an initial equilibration time of 70 ns and a production run of 150 ns.

The secondary structure analysis tool of VMD^72^ was applied on the last 100 ns of production run to obtain the secondary structure for each residue in a given frame. The helicity plot was subsequently created by calculating the ratio of frames classified as a helix divided by the total number of frames for each residue.

The area plot for the inserted proteins were obtained using g-lomepro^73^. We used 100 ns production run after an initial equilibration of 100 ns. Since the protein has no restrain of movement along the plane of the bilayer during the simulation, the frames had to be centered around the H_0_ helix center of mass.

### Transferrin-uptake assay

RPE cells expressing epsin mutants were serum-starved for 4 h. Cells were subjected to hypo- and iso-osmotic shock for 10 min and then allowed to uptake transferrin Alexa 647 (25 µg/ml) (Thermo Fisher) for a further 10 min, followed by acid wash (acetic acid buffer pH 3.0) and immediate fixation with 4% paraformaldehyde (Electron Microscopy Sciences) in PBS for 10 min. For imaging surface receptor-ligand co-localization, acid wash was not performed. The intensity of transferrin Alexa Fluor 647 was calculated using epifluorescence signal and normalized to bulk mCherry-clathrin intensity.

### Western blot

RPE cells were lysed with RIPA buffer (Thermo Fisher) containing protease inhibitor on ice for 10 min. 25 µL of lysate mixed with sample buffer (1:1 ratio) (Bio-Rad) was loaded per lane in a 10% SDS-PAGE gel (Bio-Rad). The proteins were transferred to nitrocellulose membrane and blocked for 1 h with 3% bovine serum albumin solution (in PBS). Primary antibodies against epsin (Abcam: ab75879 (1:500 dilution)), CHC (Abcam; ab2731 (1:500 dilution)), α-adaptin (Abcam: ab2807 (1:100 dilution)) were used to quantify the expression levels of the respective proteins in epsin overexpressed cells, epsin/CHC/α-adaptin knockdown cells and wild type cells. Blots with primary antibody and 1:1000 dilution of anti-GAPDH antibody were incubated overnight followed by 1 h incubation of secondary antibody conjugated to Dylight 680 nm or Dylight 800 nm. The blots were imaged using a LiCor imaging system or an Azure imaging system.

### Statistics and reproducibility

For intensity, lifetime, and initiation density data, the statistical significance was verified by one-way ANOVA test. Subsequently, Bonferroni and Holm’s multiple comparison test was performed post hoc between data while considering individual pairs to determine the *p* values. *, **, and *** were assigned to *p* < 0.05, *p* < 0.01, and *p* < 0.001, respectively.

The experiments were performed at least three times with data collected from multiple cells in each replicate, unless specified otherwise. Number of cells considered for the analysis along with the number of CCP tracks is provided in the corresponding figure captions.

### Reporting summary

Further information on research design is available in the Nature Research Reporting Summary linked to this article.

## Supplementary information

Supplementary Information

Description of Additional Supplementary Files

Supplemental Movie 1

Reporting Summary

## Data Availability

Single data points in Figs 3b and 6c are available in Supplementary Data 1. Other data that support the findings of this study are available from the corresponding author upon request.

## Code availability

The computational code can be made available upon request to A.A.

## References

[1] Kaksonen M, Roux AAA (2018). Mechanisms of clathrin-mediated endocytosis. Nat. Rev. Mol. Cell Biol..

[2] Ferguson JP (2016). Deciphering dynamics of clathrin-mediated endocytosis in a living organism. J. Cell Biol..

[3] McMahon HT, Boucrot E (2011). Molecular mechanism and physiological functions of clathrin-mediated endocytosis. Nat. Rev. Mol. Cell Biol..

[4] Conner SD, Schmid SL (2003). Regulated portals of entry into the cell. Nature.

[5] Jarsch IK, Daste F, Gallop JL (2016). Membrane curvature in cell biology: an integration of molecular mechanisms. J. Cell Biol..

[6] Cocucci E, Aguet F, Boulant S, Kirchhausen T (2012). The first five seconds in the life of a clathrin-coated pit. Cell.

[7] Schmid EM, McMahon HT (2007). Integrating molecular and network biology to decode endocytosis. Nature.

[8] Bucher D (2018). Clathrin-adaptor ratio and membrane tension regulate the flat-to-curved transition of the clathrin coat during endocytosis. Nat. Commun.

[9] Scott B (2018). Membrane bending occurs at all stages of clathrin-coat assembly and defines endocytic dynamics. Nat. Commun.

[10] Hassinger JE, Oster G, Drubin DG, Rangamani P (2017). Design principles for robust vesiculation in clathrin-mediated endocytosis. Proc. Natl Acad. Sci. USA.

[11] Walani N, Torres J, Agrawal A (2015). Endocytic proteins drive vesicle growth via instability in high membrane tension environment. Proc. Natl Acad. Sci. USA.

[12] Boulant S, Kural C, Zeeh J-CC, Ubelmann F, Kirchhausen T (2011). Actin dynamics counteract membrane tension during clathrin-mediated endocytosis. Nat. Cell Biol..

[13] Ferguson JP (2017). Mechanoregulation of clathrin-mediated endocytosis. J. Cell Sci..

[14] Joseph, J. G. & Liu, A. P. Mechanical regulation of endocytosis: new insights and recent advances. *Adv. Biosyst*. 10.1002/adbi.201900278 (2020). 10.1002/adbi.202000155 33179871

[15] Boulant, S. Assaying the contribution of membrane tension to clathrin-mediated endocytosis. in *Clathrin-Mediated Endocytosis* 37–50. 10.1007/978-1-4939-8719-1_4 (Springer New York, 2018). 10.1007/978-1-4939-8719-1_4 30129008

[16] McMahon HT (2002). Curvature of clathrin-coated pits driven by epsin. Nature.

[17] Messa M (2014). Epsin deficiency impairs endocytosis by stalling the actin-dependent invagination of endocytic clathrin-coated pits. Elife.

[18] Legendre-Guillemin V, Wasiak S, Hussain NK, Angers A, McPherson PS (2004). ENTH/ANTH proteins and clathrin-mediated membrane budding. J. Cell Sci..

[19] Holkar SS, Kamerkar SC, Pucadyil TJ (2015). Spatial control of epsin-induced clathrin assembly by membrane curvature. J. Biol. Chem..

[20] Sen A, Madhivanan K, Mukherjee D, Claudio Aguilar R (2012). The epsin protein family: coordinators of endocytosis and signaling. Biomol. Concepts.

[21] Smith SM, Baker M, Halebian M, Smith CJ (2017). Weak molecular interactions in clathrin-mediated endocytosis. Front. Mol. Biosci..

[22] Busch DJ (2015). Intrinsically disordered proteins drive membrane curvature. Nat. Commun..

[23] Stachowiak JC (2012). Membrane bending by protein–protein crowding. Nat. Cell Biol..

[24] Gleisner M (2016). Epsin N-terminal homology domain (ENTH) activity as a function of membrane tension. J. Biol. Chem..

[25] Hutchison JB, Mudiyanselage APKKK, Weis RM, Dinsmore AD (2016). Osmotically-induced tension and the binding of N-{BAR} protein to lipid vesicles. Soft Matter.

[26] Capraro BR, Yoon Y, Cho W, Baumgart T (2010). Curvature sensing by the epsin N-terminal homology domain measured on cylindrical lipid membrane tethers. J. Am. Chem. Soc..

[27] Cui H, Lyman E, Voth GA (2011). Mechanism of membrane curvature sensing by amphipathic helix containing proteins. Biophys. J..

[28] Drin G, Antonny B (2010). Amphipathic helices and membrane curvature. FEBS Lett..

[29] Shi Z, Baumgart T (2015). Membrane tension and peripheral protein density mediate membrane shape transitions. Nat. Commun..

[30] Tan X, Heureaux J, Liu AP (2015). Cell spreading area regulates clathrin-coated pit dynamics on micropatterned substrate. Integr. Biol..

[31] Irajizad E, Walani N, Veatch SL, Liu AP, Agrawal A (2017). Clathrin polymerization exhibits high mechano-geometric sensitivity. Soft Matter.

[32] Loerke D (2009). Cargo and dynamin regulate clathrin-coated pit maturation. PLoS Biol..

[33] Rosselli-Murai LK (2018). Loss of PTEN promotes formation of signaling-capable clathrin-coated pits. J. Cell Sci.

[34] Rosselli-Murai, L. K., Joseph, J. G., Lopes-Cendes, I., Liu, A. P. & Murai, M. J. The Machado–Joseph disease-associated form of ataxin-3 impacts dynamics of clathrin-coated pits. *Cell Biol. Int*. 10.1002/cbin.11312 (2020). 10.1002/cbin.11312 31970864

[35] Aguet F, Antonescu CN, Mettlen M, Schmid SL, Danuser G (2013). Advances in analysis of low signal-to-noise images link dynamin and AP2 to the functions of an endocytic checkpoint. Dev. Cell.

[36] Grassart A (2014). Actin and dynamin2 dynamics and interplay during clathrin-mediated endocytosis. J. Cell Biol..

[37] Cocucci E, Gaudin R, Kirchhausen T (2014). Dynamin recruitment and membrane scission at the neck of a clathrin-coated pit. Mol. Biol. Cell.

[38] Willy, N. M. et al. Endocytic clathrin coats develop curvature at early stages of their formation. *bioRxiv*10.1101/715219 (2019). 10.1016/j.devcel.2021.10.019 PMC11414472 34774130

[39] Wen PJ (2016). Actin dynamics provides membrane tension to merge fusing vesicles into the plasma membrane. Nat. Commun.

[40] Pietuch A, Brückner BR, Janshoff A (2013). Membrane tension homeostasis of epithelial cells through surface area regulation in response to osmotic stress. Biochim. Biophys. Acta.

[41] Wrobel LK (2002). Micropatterning tractional forces in living cells. Cell Motil. Cytoskeleton.

[42] Gauthier NC, Fardin MA, Roca-Cusachs P, Sheetz MP (2011). Temporary increase in plasma membrane tension coordinates the activation of exocytosis and contraction during cell spreading. Proc. Natl Acad. Sci. USA.

[43] Loh J (2019). An acute decrease in plasma membrane tension induces macropinocytosis via {PLD}2 activation. J. Cell Sci..

[44] Lai C-LL (2012). Membrane binding and self-association of the epsin N-terminal homology domain. J. Mol. Biol..

[45] Zeno WF (2018). Synergy between intrinsically disordered domains and structured proteins amplifies membrane curvature sensing. Nat. Commun..

[46] Wiggins P, Phillips R (2004). Analytic models for mechanotransduction: gating a mechanosensitive channel. Proc. Natl Acad. Sci. USA.

[47] Wiggins P, Phillips R (2005). Membrane-protein interactions in mechanosensitive channels. Biophys. J..

[48] Taylor MJ, Perrais D, Merrifield CJ (2011). A high precision survey of the molecular dynamics of mammalian clathrin-mediated endocytosis. PLoS Biol..

[49] Drake MT, Downs MA, Traub LM (2002). Epsin binds to clathrin by associating directly with the clathrin-terminal domain. J. Biol. Chem..

[50] Avinoam O, Schorb M, Beese CJ, Briggs JAGG, Kaksonen M (2015). Endocytic sites mature by continuous bending and remodeling of the clathrin coat. Science.

[51] Sochacki, K. A. et al. The structure and spontaneous curvature of clathrin lattices at the plasma membrane. *bioRxiv*10.1101/2020.07.18.207258 (2020). 10.1016/j.devcel.2021.03.017 PMC8081270 33823128

[52] Sinha B (2011). Cells respond to mechanical stress by rapid disassembly of caveolae. Cell.

[53] Brady RJ, Wen Y, O’Halloran TJ (2008). The ENTH and C-terminal domains of dictyostelium epsin cooperate to regulate the dynamic interaction with clathrin-coated pits. J. Cell Sci..

[54] Claudio Aguilar R, Watson HA, Wendland B (2003). The yeast epsin Ent1 is recruited to membranes through multiple independent interactions. J. Biol. Chem..

[55] Aguilar RC (2006). Epsin N-terminal homology domains perform an essential function regulating Cdc42 through binding Cdc42 GTPase-activating proteins. Proc. Natl Acad. Sci. USA.

[56] Wendland B, Steece KE, Emr SD (1999). Yeast epsins contain an essential N-terminal ENTH domain, bind clathrin and are required for endocytosis. EMBO J..

[57] Bieling P (2016). Force feedback controls motor activity and mechanical properties of self-assembling branched actin networks. Cell.

[58] Panchal SC, Kaiser DA, Torres E, Pollard TD, Rosen MK (2003). A conserved amphipathic helix in WASP/scar proteins is essential for activation of Arp2/3 complex. Nat. Struct. Biol..

[59] Cui H (2013). Understanding the role of amphipathic helices in N-bar domain driven membrane remodeling. Biophys. J..

[60] Simunovic M, Evergren E, Callan-Jones A, Bassereau P (2019). Curving cells inside and out: roles of BAR domain proteins in membrane shaping and its cellular implications. Annu. Rev. Cell Dev. Biol..

[61] Ford MGJ (2001). Simultaneous binding of PtdIns (4,5) P2 and clathrin by AP180 in the nucleation of clathrin lattices on membranes. Science.

[62] Fernandes F (2008). Role of helix 0 of the N-BAR domain in membrane curvature generation. Biophys. J..

[63] Golani G, Ariotti N, Parton RG, Kozlov MM (2019). Membrane curvature and tension control the formation and collapse of caveolar superstructures. Dev. Cell.

[64] Saffarian S, Cocucci E, Kirchhausen T (2009). Distinct dynamics of endocytic clathrin-coated pits and coated plaques. PLoS Biol..

[65] Henne WM (2010). FCHo proteins are nucleators of clathrin-mediated endocytosis. Science.

[66] Kelly BT (2014). AP2 controls clathrin polymerization with a membrane-activated switch. Science.

[67] Chen C, Zhuang X (2008). Epsin 1 is a cargo-specific adaptor for the clathrin-mediated endocytosis of the influenza virus. Proc. Natl Acad. Sci. USA.

[68] Chen H, De Camilli P (2005). The association of epsin with ubiquitinated cargo along the endocytic pathway is negatively regulated by its interaction with clathrin. Proc. Natl Acad. Sci. USA.

[69] Tinevez JY (2017). TrackMate: an open and extensible platform for single-particle tracking. Methods.

[70] Huang J, MacKerell AD (2013). CHARMM36 all-atom additive protein force field: validation based on comparison to NMR data. J. Comput. Chem..

[71] Jo S, Kim T, Iyer VG, Im W (2008). CHARMM-GUI: a web-based graphical user interface for CHARMM. J. Comput. Chem..

[72] Gapsys V, De Groot BL, Briones R (2013). Computational analysis of local membrane properties. J. Comput. Aided Mol. Des..

[73] Humphrey W, Dalke A, Schulten K (1996). VMD: visual molecular dynamics. J. Mol. Graph..

