# Supplementary material for: Complimentary action of structured and unstructured domains of epsin supports clathrin-mediated endocytosis at high tension
Source: Commun Biol. 2020 Dec 8;3:743. doi: 10.1038/s42003-020-01471-6 (PMC7722716; doi:10.1038/s42003-020-01471-6)
Supplement: Supplementary file 1 — Supplementary Information [file 42003_2020_1471_MOESM1_ESM.pdf]

**Supplemental Figures**

# Complimentary action of structured and unstructured domains of epsin supports clathrin-mediated endocytosis at high tension

Jophin G. Joseph[1], Carlos Osorio[2], Vivian Yee[1], Ashutosh Agrawal[2], Allen P. Liu[1,3,4,5]

[1] Department of Mechanical Engineering, University of Michigan, Ann Arbor, Michigan, USA

[2] Department of Mechanical Engineering, University of Houston, Houston, Texas, USA

[3] Department of Biomedical Engineering, University of Michigan, Ann Arbor, Michigan, USA

[4] Cellular and Molecular Biology Program, University of Michigan, Ann Arbor, Michigan, USA

[5] Department of Biophysics, University of Michigan, Ann Arbor, Michigan, USA

Corresponding author:

A.P.L: allenliu@umich.edu; 2350 Hayward Street, University of Michigan, Ann Arbor, Michigan 48109. Tel: +1 734-764-7719.

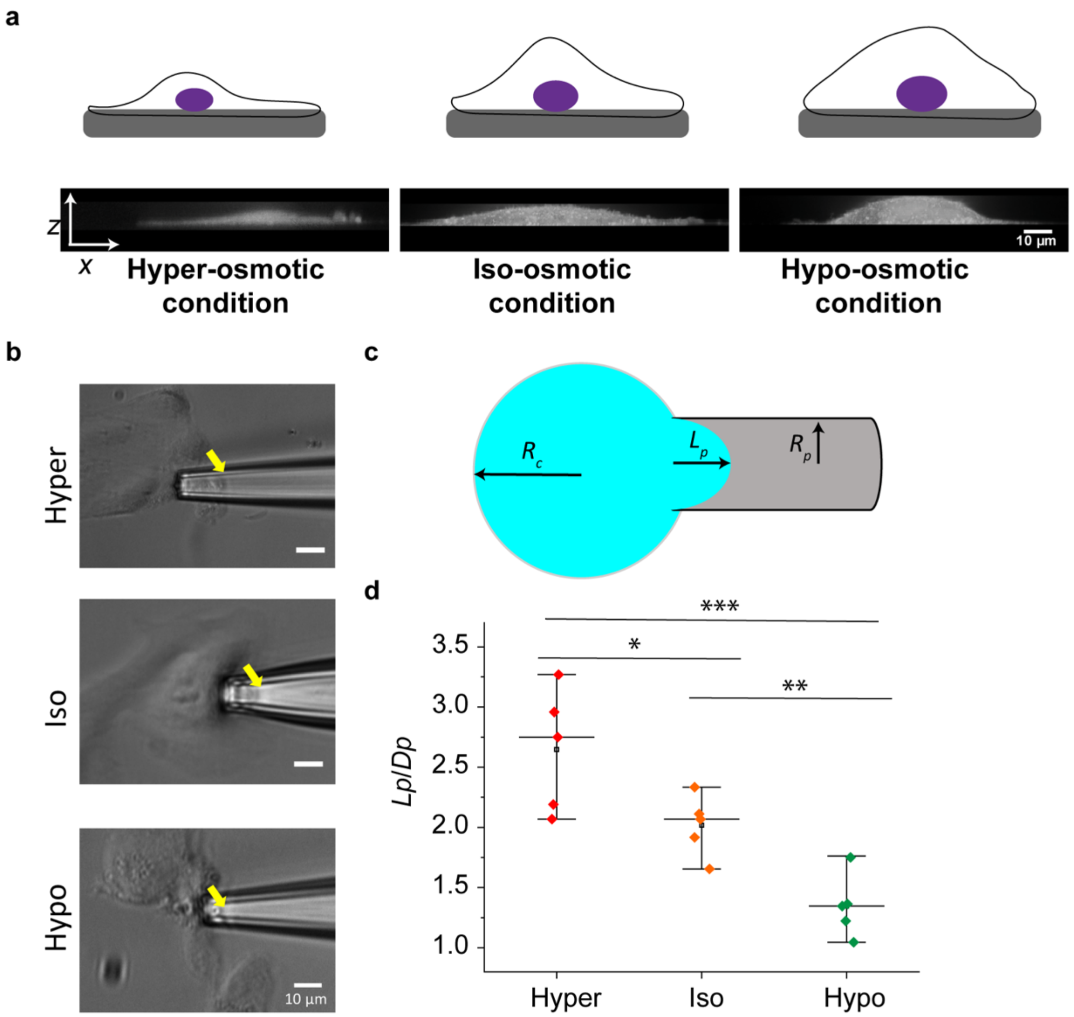


**Supplementary Figure 1. Micropipette aspiration shows osmotic shock increases tension. a.** Osmotic shock experiment for acute tension drop and spike, as described in the Materials and Methods. Representative confocal cross section images reconstructed from 3D image stacks of cells under hyper-, iso- and hypo-osmotic conditions. **b.** Plasma membrane aspirated into the micropipette due to negative hydrostatic pressure ($\Delta P$ = -2.16 kPa) at hyper-, iso- and hypo-osmotic conditions. **c.** The simplified schematic of plasma membrane aspiration into the micropipette. $L_p$ is the total length of aspiration, $R_c$ is the radius of the cell (assuming spherical shape) and $R_p$ is the radius of the pipette ($D_p = 2R_p$). **c.** $L_p/D_p$ values of cells under hyper-, iso- and hypo-osmotic conditions. *, **, *** represent $p < 0.05$, $p < 0.01$, and $p < 0.001$, respectively.

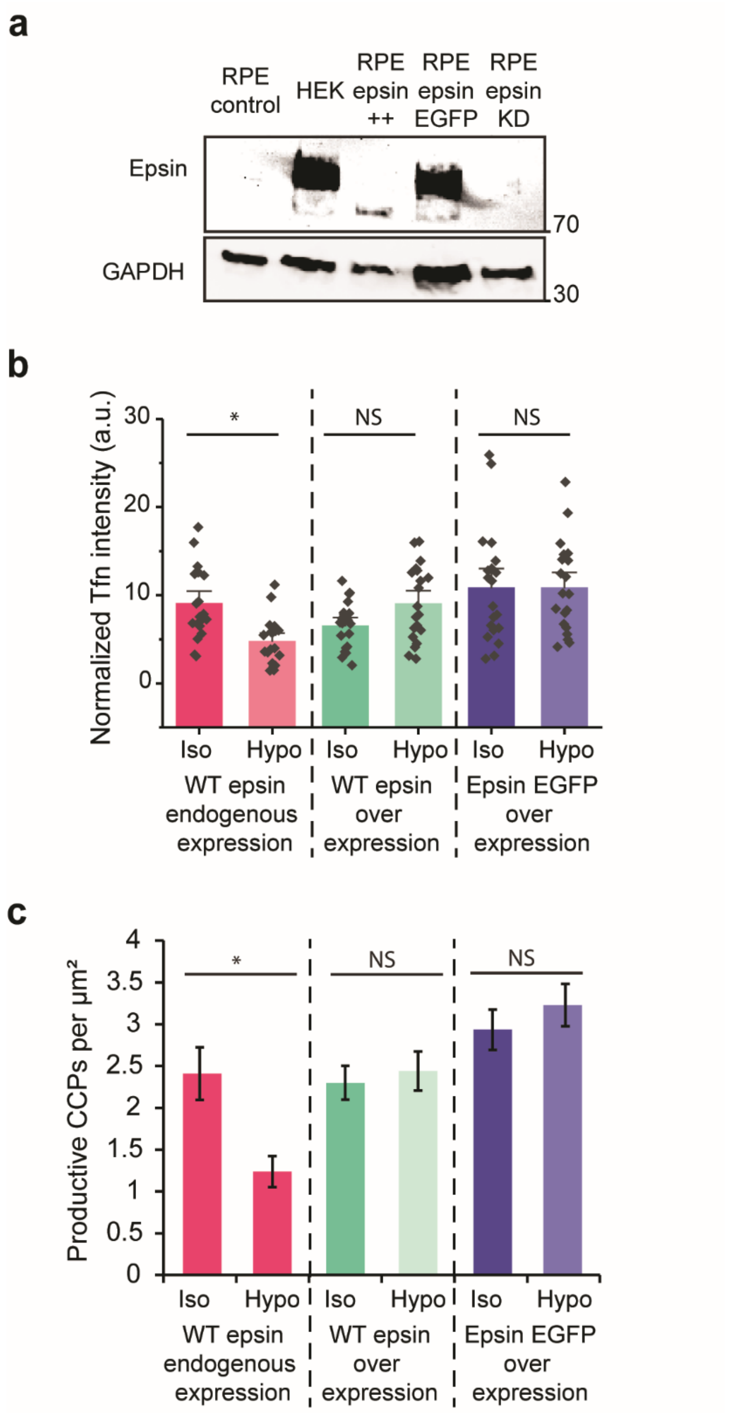


**Supplementary Figure 2. Overexpression of epsin support the formation of productive CCPs and cargo uptake at high tension. a.** Western blot showing the expression of epsin in RPE cells and HEK cells (highest epsin expression shown), RPE cells overexpressing WT epsin, epsin EGFP and knockdown of epsin, with GAPDH as a loading control. **b.** Transferrin Alexa 647 intensities in RPE cells with endogenous expression of WT epsin, overexpression of WT epsin and overexpression of epsin EGFP under iso- and hypo-osmotic conditions. The intensities were normalized with bulk mCherry clathrin intensity. The number of cells for iso- and

hypo-osmotic conditions were 20 each for WT epsin endogenous expression, WT epsin overexpression and epsin EGFP expression. The experiment was performed once. The error bars denote standard error. **c.** The number of productive CCPs per $\mu m^2$ of cell area in RPE cells with endogenous expression of WT epsin, overexpression of WT epsin and overexpression of epsin EGFP under iso- and hypo-osmotic conditions during 5-minute duration of SIM-TIRF microscopy. NS denotes not significant. *, **, *** represent $p < 0.05$, $p < 0.01$ and $p < 0.001$, respectively.

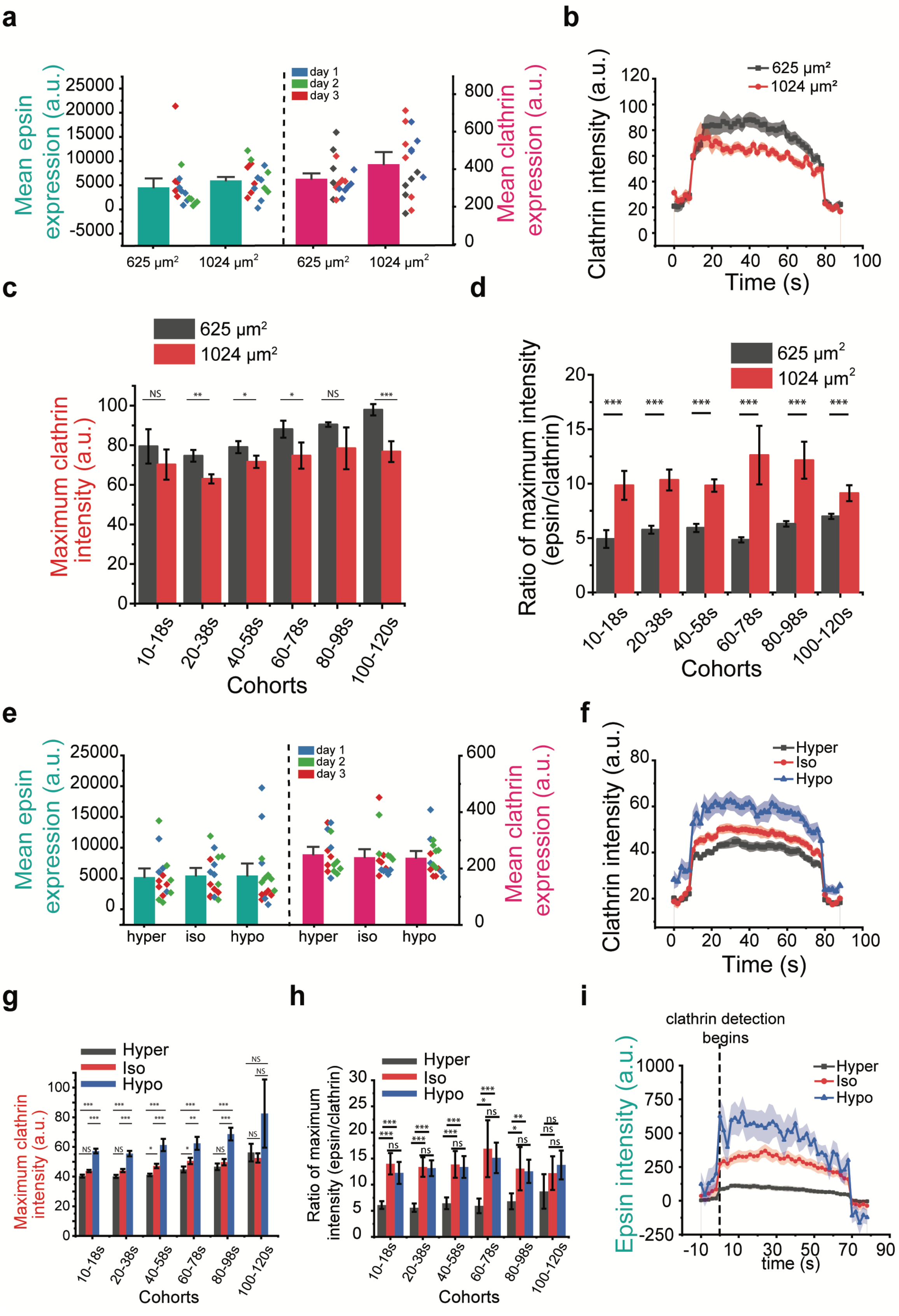
a
b
c
d
e
f
g
h
i
Mean epsin expression (a.u.)
Mean clathrin expression (a.u.)
day 1
day 2
day 3
625 µm2
1024 µm2
Clathrin intensity (a.u.)
Time (s)
Maximum clathrin intensity (a.u.)
Ratio of maximum intensity (epsin/clathrin)
Cohorts
10-18s
20-38s
40-58s
60-78s
80-98s
100-120s
hyper
iso
hypo
Hyper
Iso
Hypo
Ratio of maximum intensity (epsin/clathrin)
clathrin detection begins
Epsin intensity (a.u.)
time (s)

**Supplementary Figure 3. Clathrin recruitment into epsin-positive CCPs is distinct between increasing resting tension and acute tension increase. a**. The average bulk expression of epsin EGFP and mCherry clathrin in RPE cells used for microcontact printing experiments. Different colored points encode the day of experiment. **b.** The average intensity trace of clathrin in 25 (black) and 32 µm (red) islands for epsin-positive CCPs with 60 -78 s lifetime. **c.** The average plateau intensity of clathrin across different epsin-positive CCP lifetime cohorts. **d**. The ratio of maximum intensity of epsin EGFP and mCherry clathrin across different epsin-positive CCP lifetime cohorts for microcontact printing experiment. **e.** The mean clathrin and epsin intensities under different osmotic conditions and showing distribution of data from collected from different days. Error bars denote standard errors from the entire data set. **f**. The average bulk expression of epsin EGFP and mCherry clathrin in RPE cells used for osmotic shock experiments. Different colored points encode the day of experiment **g.** The intensity profiles of epsin-positive CCPs with 60 -78 s lifetime for different osmotic conditions. **h.** The average plateau intensity of clathrin across different epsin-positive CCP lifetime cohorts. **i.** The ratio of maximum intensity of epsin EGFP and mCherry clathrin across different epsin-positive CCP lifetime cohorts for osmotic shock experiments. **i.** The intensity profile of epsin EGFP in epsin-positive CCPs with 60 -78 s lifetime for different osmotic conditions by considering epsin EGFP as the primary detection channel. The beginning of detection of clathrin is shown with the dotted line. For a, b, c, d, $N_{cells}$ for 25 µm square and $N_{cells}$ for 32 µm square are 18 ($N_{tracks}$ = 17534) and 19 ($N_{tracks}$ = 30188), respectively. For e, f, g, h, i, the number of cells for hyper-, iso-, and hypo-osmotic conditions were 19 ($N_{tracks}$ = 38282), 19 ($N_{tracks}$= 58574), and 16 ($N_{tracks}$ = 32644), respectively. The error bars denote standard error. NS denotes not significant. *, **, *** represent $p < 0.05$, $p < 0.01$ and $p < 0.001$, respectively.

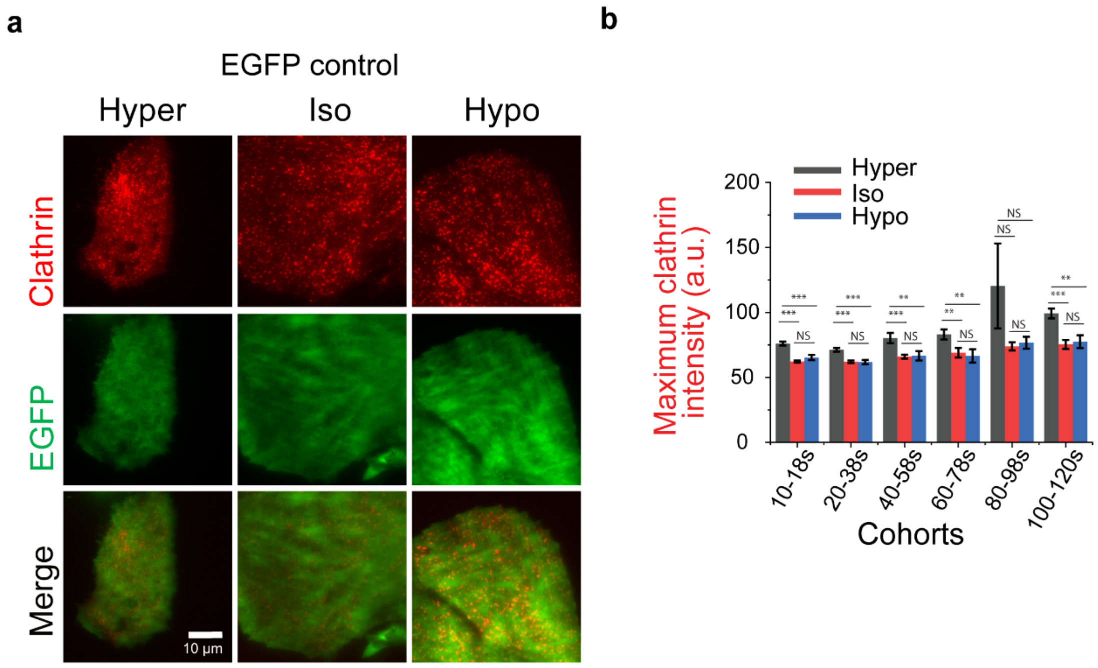


**Supplementary Figure 4. Clathrin recruitment into CCPs in EGFP control cells remains unchanged with increase in acute tension and increases during acute tension drop. a.** Representative fluorescence images of clathrin and EGFP of RPE cells expressing EGFP as a control under different osmotic conditions. **b.** The average plateau intensity of clathrin across different CCP lifetime cohorts in RPE cells. For b, $N_{cells}$ expressing EGFP for hyper-, iso-, and hypo-osmotic conditions were 7 ($N_{tracks}$= 16149), 8 ($N_{tracks}$= 14315), and 11 ($N_{tracks}$= 20134), respectively. The error bars denote standard error. NS denotes not significant. *, **, *** represent $p < 0.05$, $p < 0.01$ and $p < 0.001$, respectively.

.

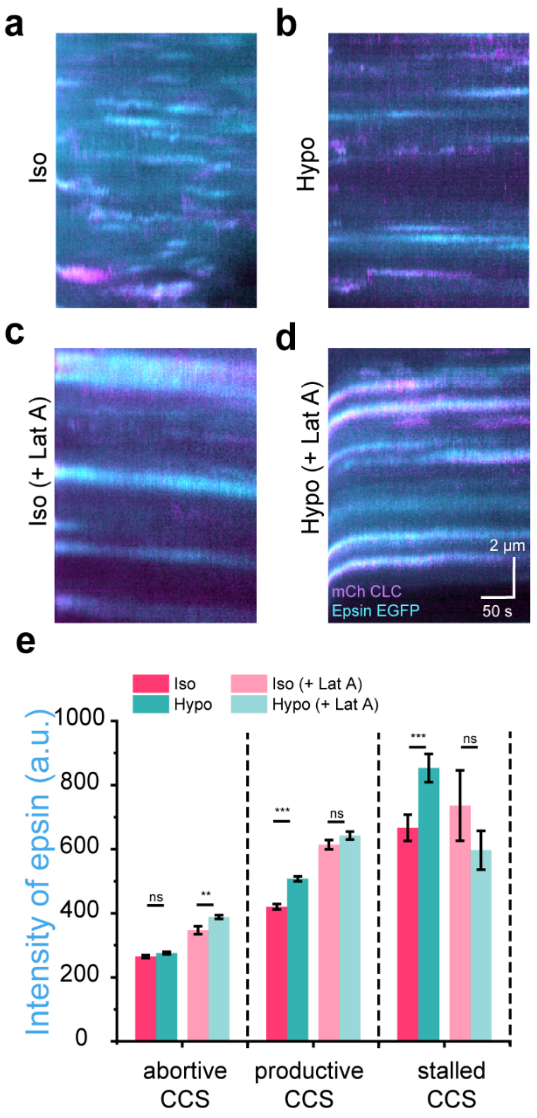


**Supplementary Figure 5. Epsin recruitment increases in CCSs under actin disruption.** Kymographs of CCSs in RPE cells overexpressing epsin EGFP (cyan) and mCherry clathrin (magenta) under, **a.** iso-osmotic condition, **b.** hypo-osmotic condition, **c.** iso-osmotic condition and 0.5 μM Latrunculin A treatment, **d.** hypo-osmotic condition and 0.5 μM Lat A treatment. **e.** Intensity of epsin for abortive, productive and stalled CCSs under iso-osmotic condition and hypo-osmotic condition with and without Latrunculin A treatment. For e, the number of cells for iso-, hypo-osmotic conditions without Latrunculin A and iso-, hypo-osmotic conditions with Latrunculin A were 12 ($N_{tracks}$=15235),12 ($N_{tracks}$=14147) and 12 ($N_{tracks}$= 15640), 12 ($N_{tracks}$= 19360) respectively. The error bars denote standard error. NS denotes not significant. *, **, *** represent $p < 0.05$, $p < 0.01$ and $p < 0.001$, respectively.

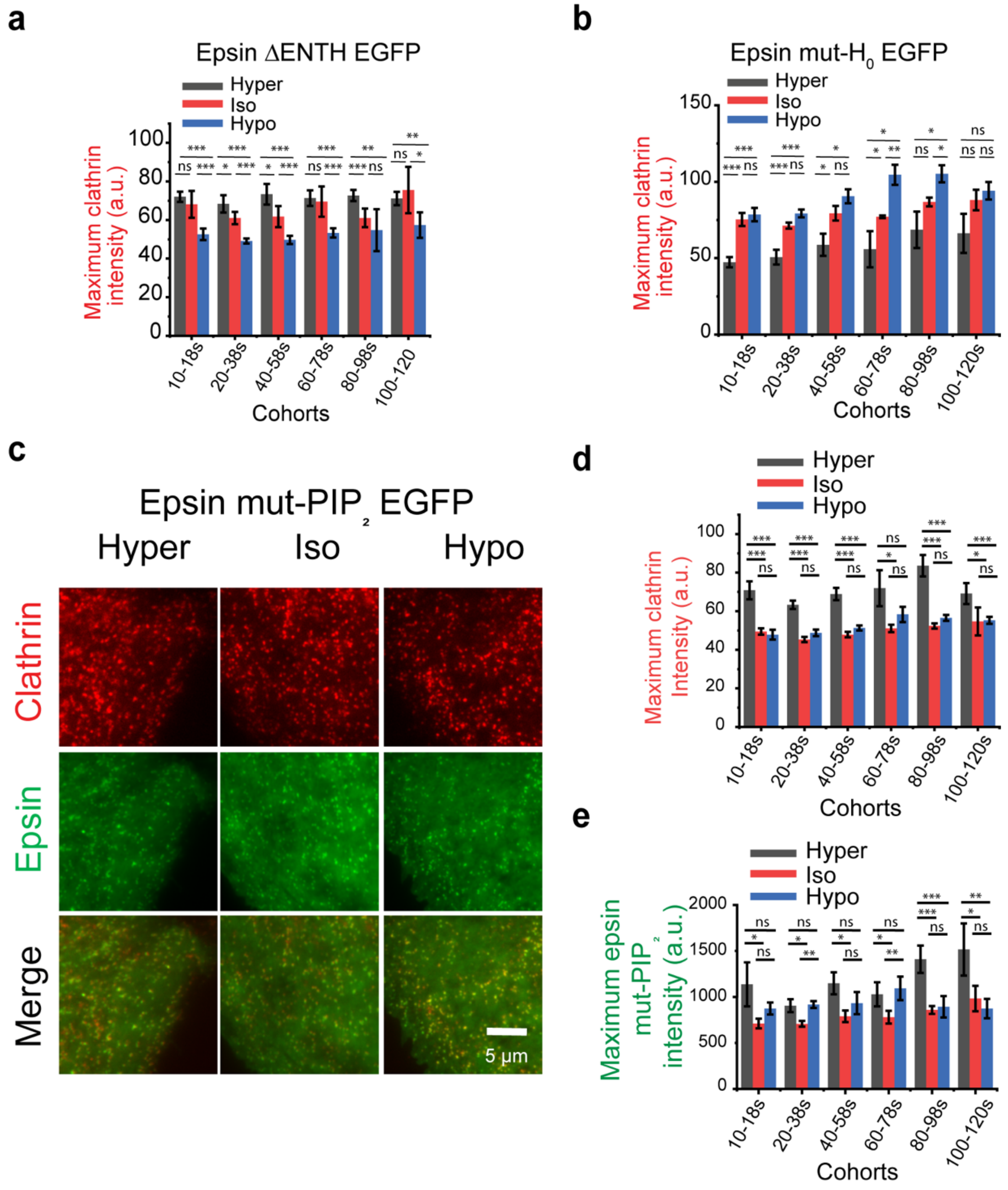


**Supplementary Figure 6. Recruitment trends of clathrin into CCPs due acute membrane tension change are not altered significantly by overexpression of epsin mutants lacking a functioning ENTH domain. a.** The average plateau intensity of clathrin in different epsin ΔENTH-positive CCP lifetime cohorts in different osmotic conditions. **b.** The average plateau intensity of clathrin in different epsin mut-$H_0$ positive CCP lifetime cohorts in different osmotic

conditions. **c.** Representative fluorescence images of clathrin and epsin mut-$PIP_2$ of RPE cells expressing epsin mut-$PIP_2$ EGFP under different osmotic conditions. **d.** The average plateau intensity of clathrin in different epsin mut-$PIP_2$ positive CCP lifetime cohorts in different osmotic conditions. **e.** The average plateau intensity of epsin mut-$PIP_2$ in different epsin mut-$PIP_2$ positive CCP lifetime cohorts in different osmotic conditions. The $N_{cells}$ expressing epsin ΔENTH EGFP for hyper-, iso-, and hypo-osmotic conditions in a. were 12 ($N_{tracks}$= 19328), 12 ($N_{tracks}$= 20078), and 12 ($N_{tracks}$= 31952), respectively. The $N_{cells}$ expressing epsin mut-$H_0$ for hyper-, iso-, and hypo-osmotic conditions in b. were 18 ($N_{tracks}$= 32327), 19 ($N_{tracks}$= 33354), and 18 ($N_{tracks}$= 40602), respectively. The $N_{cells}$ expressing epsin mut-$PIP_2$ for hyper-, iso-, and hypo-osmotic conditions in c, d and e. were 8, 8, and 8, respectively. The error bars denote standard error. NS denotes not significant. *, **, *** represent $p < 0.05$, $p < 0.01$ and $p < 0.001$, respectively.

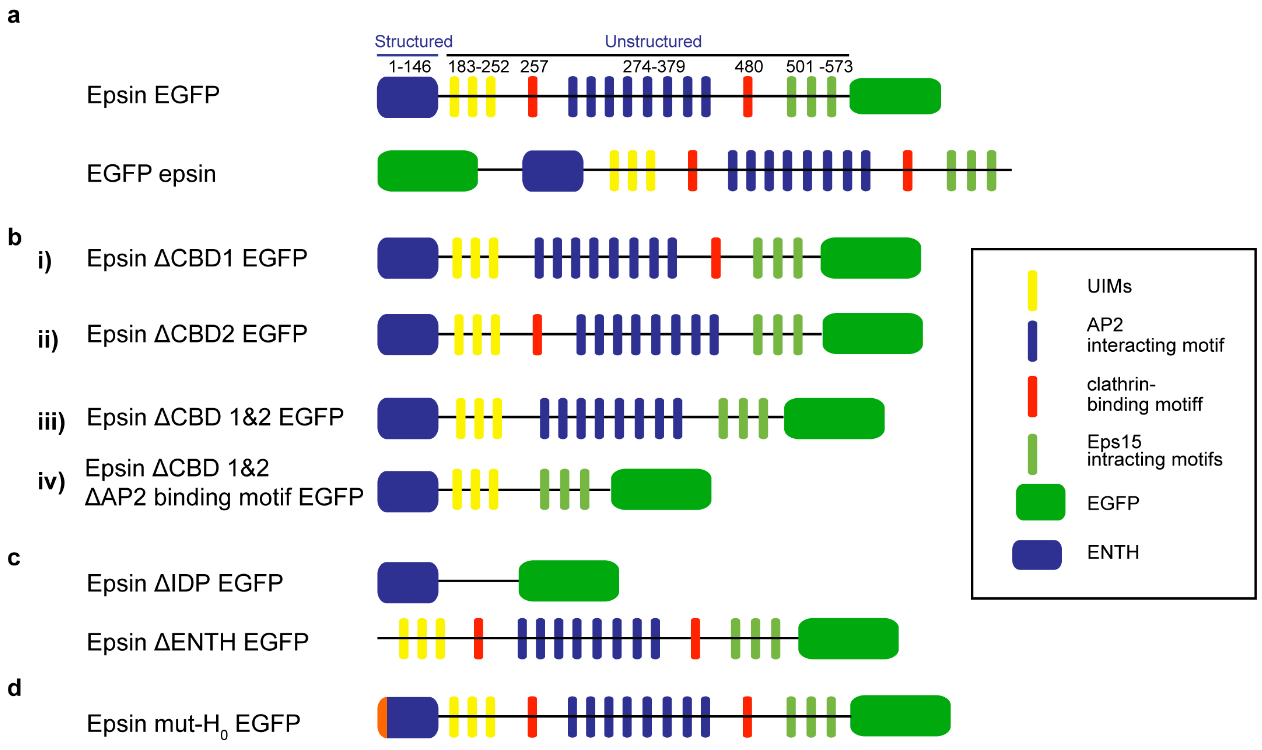


**Supplementary Figure 7. Epsin mutants. a.** Full length epsin with EGFP tagged on C-terminus and N-terminus. Different domains/motifs of epsin and their location is shown. **b**. Epsin EGFP mutants generated by deleting binding domains (i) clathrin-binding domain1 (CBD1), (ii) clathrin-binding domain 2 (CBD2), (iii) clathrin-binding domain 1 and 2 (CBD1 & 2), (iv) CBD1 & 2 and region containing repeated DPW motifs binding to AP2. **c.** Epsin mutant with entire unstructured region of the protein deleted (ΔIDP) and epsin with entire structured region of the protein deleted (ΔENTH). **d**. Epsin EGFP after mutating $H_0$ helix in the N-terminus of protein to alanine residues.

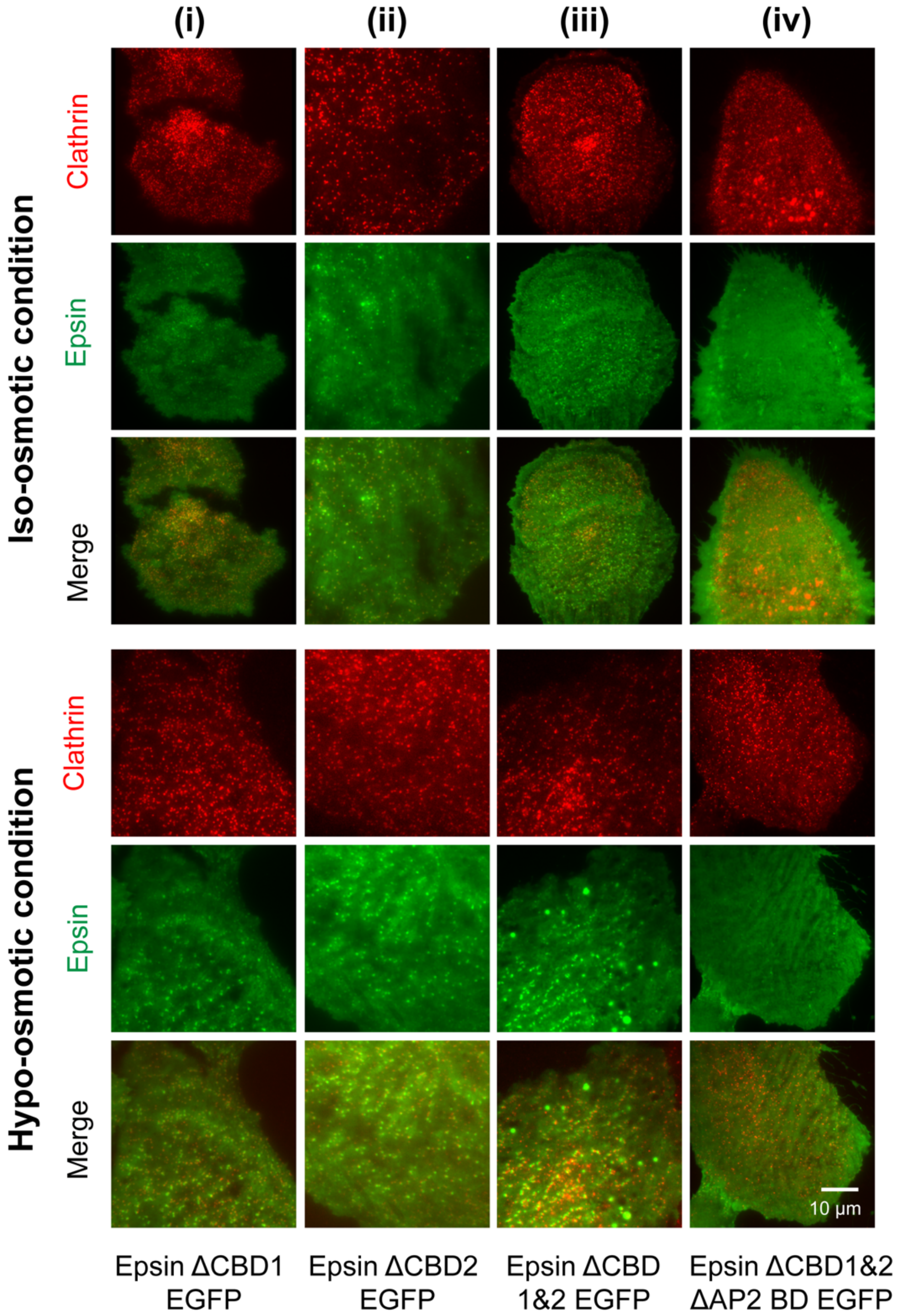


**Supplementary Figure 8. Removal of AP2 and clathrin binding sites render epsin cytosolic.** Representative fluorescence images of cells overexpressing EGFP-tagged epsin and epsin mutants under iso- (top panel) and hypo- (bottom panel) osmotic conditions**.** Cells overexpressing (i) epsin ΔCBD1 EGFP, (ii) epsin ΔCBD2 EGFP, (iii) epsin ΔCBD1&2 EGFP, (iv) epsin ΔCBD1&2 and ΔAP2 binding domains EGFP along mCherry CLC is shown.

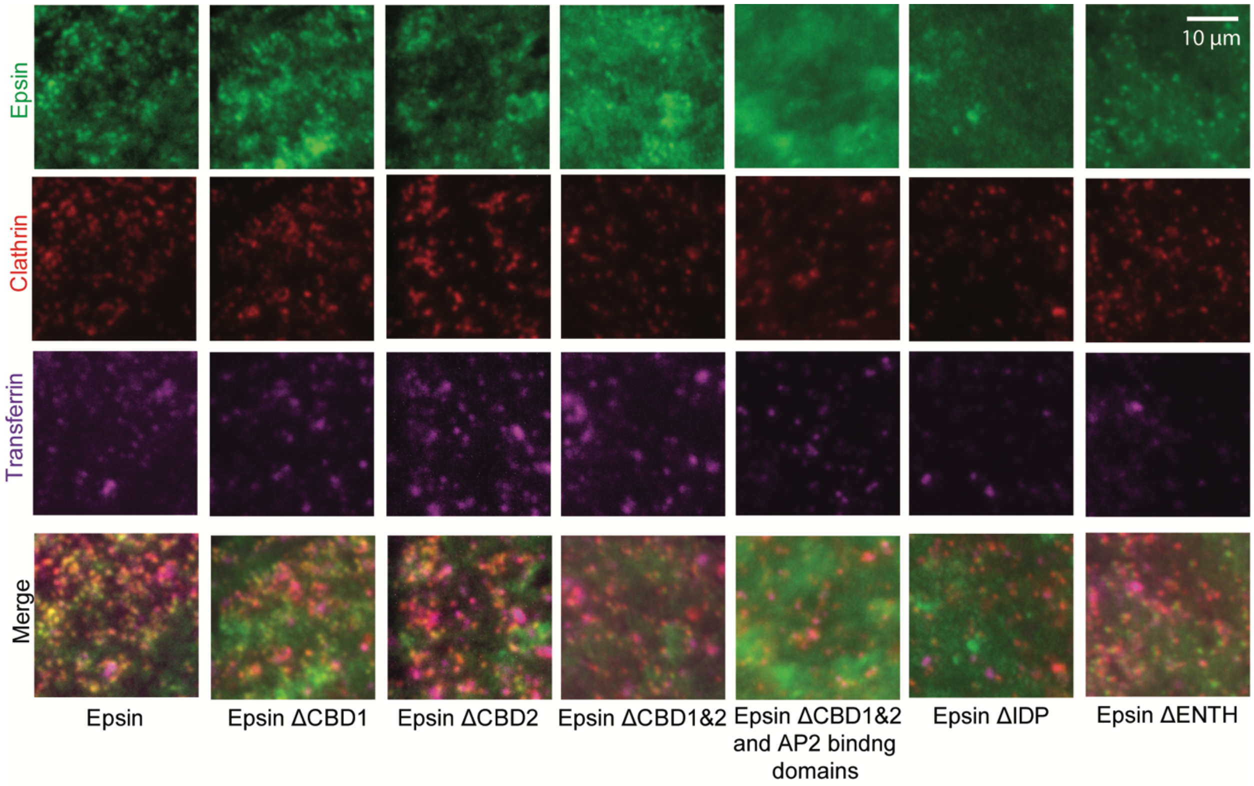


**Supplementary Figure 9. Transferrin uptake in cells overexpressing EGFP-tagged epsin and epsin mutants.** Representative fluorescence images of cells expressing epsin EGFP, epsin ΔCBD1 EGFP, epsin ΔCBD2 EGFP, epsin ΔCBD1&2 EGFP, epsin ΔCBD1&2 and ΔAP2 binding domains EGFP, epsin ΔIDP EGFP, epsin ΔENTH EGFP, along with mCherry CLC and Alexa Fluor 647 transferrin (25 µg/ml with 10 min incubation).

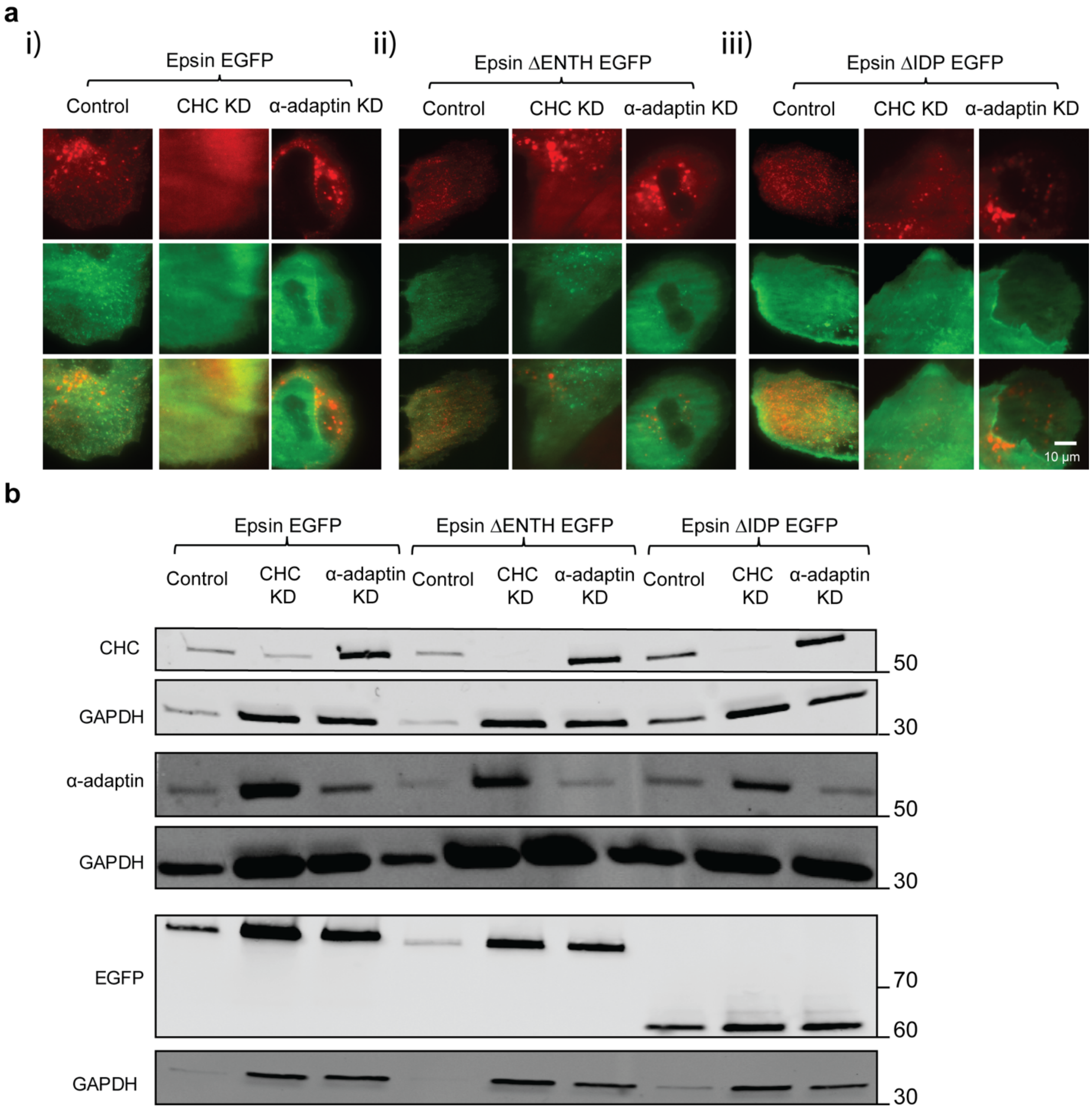


**Supplementary Figure 10. Knocking down clathrin heavy chain or AP2 subunit disrupts the recruitment of epsin**. **a.** Representative fluorescence images of (i) full length epsin EGFP, (ii) epsin ΔENTH EGFP and (iii) epsin ΔIDP EGFP, along with mCherry CLC in RPE cells with control (scramble shRNA), clathrin heavy chain (CHC) shRNA knockdown, and α-adaptin shRNA knockdown. **b.** Western blot showing the knockdown of CHC and α-adaptin, with GAPDH as a loading control. EGFP panel shows the expression of fusion protein epsin EGFP, epsin ΔENTH EGFP, and epsin ΔIDP EGFP.
