# Supplementary material for: Complimentary action of structured and unstructured domains of epsin supports clathrin-mediated endocytosis at high tension
Source: Commun Biol. 2020 Dec 8;3:743. doi: 10.1038/s42003-020-01471-6 (PMC7722716; doi:10.1038/s42003-020-01471-6)
Supplement: Supplementary file 2 — Description of Additional Supplementary Files [file 42003_2020_1471_MOESM2_ESM.pdf]

## Description of Additional Supplementary Files

File Name: Supplementary Data 1

Description: Initiation density values of epsin EGFP and epsin del ENTH EGFP for iso- and hypo-osmotic conditions are provided. Fraction of productive CCPs with and without EGFP-tagged protein recruitment in cells overexpressing epsin EGFP, epsin del ENTH EGFP and EGFP (control) for hyper-, iso-, and hypo-osmotic condition are provided.

File Name: Supplementary Movie 1

Description: The formation of abortive, productive and stalled CCPs in RPE cells captured using SIM-TIRF. CCPs recruiting epsin EGFP are shown.
