# Supplementary material for: Complimentary action of structured and unstructured domains of epsin supports clathrin-mediated endocytosis at high tension
Source: Commun Biol. 2020 Dec 8;3:743. doi: 10.1038/s42003-020-01471-6 (PMC7722716; doi:10.1038/s42003-020-01471-6)
Supplement: Supplementary file 4 — Reporting Summary [file 42003_2020_1471_MOESM4_ESM.pdf]

nature research

Corresponding author(s): Allen P. Liu

Last updated by author(s): Oct 18, 2020

Reporting Summary

Nature Research wishes to improve the reproducibility of the work that we publish. This form provides structure for consistency and transparency in reporting. For further information on Nature Research policies, see Authors & Referees and the Editorial Policy Checklist .

Statistics

For all statistical analyses, confirm that the following items are present in the figure legend, table legend, main text, or Methods section.

n/a	Confirmed	
☐	☒	The exact sample size (*n*) for each experimental group/condition, given as a discrete number and unit of measurement
☐	☒	A statement on whether measurements were taken from distinct samples or whether the same sample was measured repeatedly
☐	☒	The statistical test(s) used AND whether they are one- or two-sided<br>*Only common tests should be described solely by name; describe more complex techniques in the Methods section.*
☒	☐	A description of all covariates tested
☐	☒	A description of any assumptions or corrections, such as tests of normality and adjustment for multiple comparisons
☐	☒	A full description of the statistical parameters including central tendency (e.g. means) or other basic estimates (e.g. regression coefficient) AND variation (e.g. standard deviation) or associated estimates of uncertainty (e.g. confidence intervals)
☐	☒	For null hypothesis testing, the test statistic (e.g. *F*, *t*, *r*) with confidence intervals, effect sizes, degrees of freedom and *P* value noted<br>*Give P values as exact values whenever suitable.*
☒	☐	For Bayesian analysis, information on the choice of priors and Markov chain Monte Carlo settings
☒	☐	For hierarchical and complex designs, identification of the appropriate level for tests and full reporting of outcomes
☒	☐	Estimates of effect sizes (e.g. Cohen's *d*, Pearson's *r*), indicating how they were calculated

Our web collection on statistics for biologists contains articles on many of the points above.

Software and code

Policy information about availability of computer code

Data collection	Micro-manager 1.4 (for TIRF imaging and micro-pipette imaging), NIS-Elements AR 5.02 (for SIM TIRF Imaging), MetaMorph 7.7 (for confocal imaging), Sapphire Biomolecular Imager and LiCOR Image Studio (For Western blot imaging)
Data analysis	MATLAB 2014a (Fluorescent image analysis for TIRF using cmeAnalysis pipeline), ImageJ 1.52 (SIM TIRF analysis using TrackMate v5.2), Microsoft Excel 2016 (Data organization and statistical testing), Origin Pro 2019 (Data plotting), GROMACS (Molecular Dynamics Simulations), Visual Molecular Dynamics (for secondary structure analysis), g-lomepro (for generating area plots for proteins insertion)

For manuscripts utilizing custom algorithms or software that are central to the research but not yet described in published literature, software must be made available to editors/reviewers. We strongly encourage code deposition in a community repository (e.g. GitHub). See the Nature Research guidelines for submitting code & software for further information.

Data

Policy information about availability of data

All manuscripts must include a data availability statement. This statement should provide the following information, where applicable:

- Accession codes, unique identifiers, or web links for publicly available datasets
- A list of figures that have associated raw data
- A description of any restrictions on data availability

The data that support the findings of this study are available from the corresponding author upon request. The computational code can be made available upon request to Dr. Ashutosh Agrawal.

Field-specific reporting

Please select the one below that is the best fit for your research. If you are not sure, read the appropriate sections before making your selection.

- [x] Life sciences
- [ ] Behavioural & social sciences
- [ ] Ecological, evolutionary & environmental sciences

For a reference copy of the document with all sections, see nature.com/documents/nr-reporting-summary-flat.pdf

Life sciences study design

All studies must disclose on these points even when the disclosure is negative.

Sample size	All imaging data was performed with at least n=3 experiments, with each experiment consisting of 2 cells imaged (for TIRF SIM) to 4 cells imaged (Dual color TIRF). For each condition of imaging involving clathrin-coated pits (CCPs) at least 10000, CCP were considered for making the inference. Exact number of cells considered and number of CCP tracks for each experiment is provided in the manuscript.
Data exclusions	TIRF images sets of individual cells were removed from consideration if they were severely photo bleached such that no structure is visible on inspection with human eye.
Replication	All experiments were repeated at least n=3 to show reproducibility.
Randomization	Fluorescent images of cells were acquired randomly chosen regions on a 22x22 mm #1.5 cover slip or glass bottom dish. Cells co-expressing the fluorescent protein of interest were only imaged (on visual inspection from human eye).
Blinding	Data analysis is mostly performed with computer algorithms with only initial inputs from investigators. Hence data output is not subjected to biases of the investigators.

Reporting for specific materials, systems and methods

We require information from authors about some types of materials, experimental systems and methods used in many studies. Here, indicate whether each material, system or method listed is relevant to your study. If you are not sure if a list item applies to your research, read the appropriate section before selecting a response.

Materials & experimental systems

n/a	Involved in the study
[ ]	[x] Antibodies
[ ]	[x] Eukaryotic cell lines
[x]	[ ] Palaeontology
[x]	[ ] Animals and other organisms
[x]	[ ] Human research participants
[x]	[ ] Clinical data

Methods

n/a	Involved in the study
[x]	[ ] ChIP-seq
[x]	[ ] Flow cytometry
[x]	[ ] MRI-based neuroimaging

Antibodies

Antibodies used	epsin (Abcam: ab75879 (1:500 dilution)), CHC (Abcam; ab2731 (1:500 dilution)), α-adaptin (Abcam: ab2807 (1:100 dilution))
Validation	Each antibody was purchased were validated by ABCAM.

Eukaryotic cell lines

Policy information about cell lines

Cell line source(s)	Retinal pigment epithelial (RPE) cell was a gift from Sandra Schmid (UT Southwestern Medical Centre).
Authentication	Cells were not authenticated.
Mycoplasma contamination	Cells were not tested for Mycoplasma contamination.
Commonly misidentified lines (See ICLAC register)	Not applicable
